# Non-invasive modulation of meningeal lymphatics ameliorates ageing and Alzheimer’s disease-associated pathology and cognition in mice

**DOI:** 10.1038/s41467-024-45656-7

**Published:** 2024-02-16

**Authors:** Miao Wang, Congcong Yan, Xi Li, Tianhao Yang, Shengnan Wu, Qian Liu, Qingming Luo, Feifan Zhou

**Affiliations:** 1 State Key Laboratory of Digital Medical Engineering, School of Biomedical Engineering, Hainan University, Haikou, 570100 China; 2 Key Laboratory of Brain Health Intelligent Evaluation and Intervention, Ministry of Education, School of Medical Technology, Beijing Institute of Technology, 100081 Beijing, China

**Keywords:** Alzheimer's disease, Neuroimmunology, Blood-brain barrier

## Abstract

Meningeal lymphatic vessels (mLVs) have been shown to be involved in amyloid beta (Aβ) clearance, which is considered as a potential therapeutic target for Alzheimer’s disease (AD). In this study, based on the superficial spatial distribution of mLVs, a near-infrared light is employed to modulate lymphatic drainage, significantly improving cognition of both aged and AD (5xFAD and APP/PS1) mice, and alleviating AD-associated pathology by reducing Aβ deposition, neuroinflammation and neuronal damage. Furthermore, transmission electron microscopy imaging and RNA sequencing data indicate amelioration of mitochondrial metabolism and cellular junction of meningeal lymphatic endothelial cells (mLECs) by light modulation. These studies collectively suggest that near-infrared light treatment can improve cognitive function by strengthening scavenging ability of mLVs through restoring mLEC function. In conclusion, lymphatic drainage potentiation by light promotes pathological remission and cognitive enhancement in aging and AD mouse models, which offers a potential amelioration strategy for neurodegenerative diseases.

## Abstract (web summary)

Meningeal lymphatic vessels have been associated with amyloid beta clearance, which is considered as a modulation target for Alzheimer’s disease (AD) treatment. Here, the authors show that transcranial light treatment can enhance meningeal lymphatic drainage in aged and AD model mice and improve AD-associated pathology and cognitive function.

## Introduction

For a long time, the brain has been considered immune privileged owing to the lack of lymphatic drainage system^1^. In 2015, meningeal lymphatic vessels (mLVs) located in the dura were discovered, which constitute an extensive lymphatic drainage network to remove macromolecular waste and inflammatory mediators, direct immune cell transport, and coordinate immune responses in central nervous system (CNS)^2,3^. Recent studies have revealed that mLVs system is associated with the progression of ageing, Alzheimer’s disease (AD), Parkinson’s disease (PD), traumatic brain injury (TBI), subarachnoid hemorrhage (SAH), CNS viral infection and other nervous system diseases, and the changes of mLVs transport capability can significantly affect the disease development^4–9^. These studies indicate that modulating mLVs drainage can be an effective strategy for neurologic diseases.

AD is an age-associated neurodegenerative disease with high mortality. The main pathological features of AD are β-amyloid (Aβ) abnormal aggregation and neuronal tangles in brain that contribute to neuronal dysfunction and cognitive decline^10^. MLVs have been confirmed to be functionally degenerated with ageing or AD progression, which might be an underlying factor for exacerbated cognitive dysfunction and neural impairment. Viral-mediated vascular endothelial growth factor C (VEGF-C) treatment via intracisterna magna (i.c.m.) injection could efficiently boost function of mLVs, and then enhance lymphatic drainage to clear toxic molecules in CNS, as well as improve learning and memory ability^4,5^. Therefore, mLVs might be a therapeutical target for age-associated cognitive deficits. However, for chronic and progressive neurodegenerative disease, like AD, invasive therapy models are not feasible. Altogether, the development of non-invasive treatment modality is necessary for AD treatment.

The mLVs network is superficially distributed in the dura mater, which provides a promising strategy for transcranial neuromodulation therapies to alleviate CNS diseases through mLVs drainage modulation. In this work, based on the optical window for tissue penetration, we demonstrate that near-infrared light could modulate the function of meningeal lymphatic endothelial cells (mLECs), which in turn could contribute to mLVs drainage for the remission of pathology and the enhancement of cognitive function of aged and AD mice. Our findings reveal that the enhancement on mitochondrial metabolic homeostasis of mLECs by light is determined to promote cell adhesion and growth, which possibly potentiates cell functions and mLECs junctions to further boost meningeal lymphatic transport in the CNS for pathology amelioration.

## Results

### Light modulates mLVs drainage and attenuates cognitive decline in aged mice

**Fig. 1:**
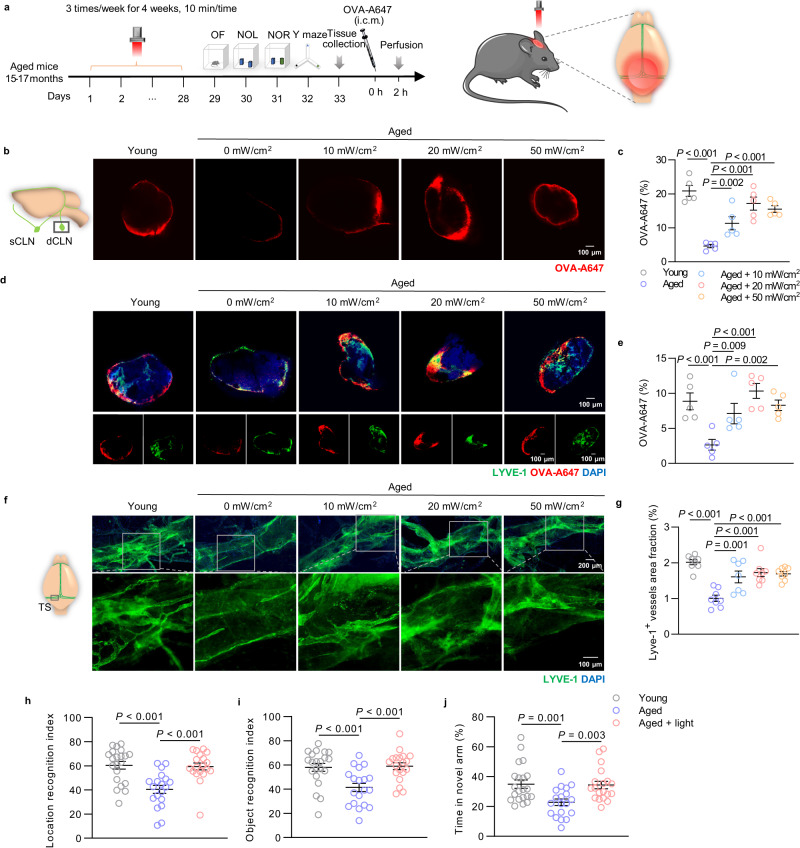
Effects of light on cognition and mLVs drainage in aged mice. **a** Schedule of treatments and behavior tests of aged mice. **b** Representative images of deep cervical lymph nodes (dCLNs) accumulated with OVA-A647 at 2 h after injection (i.c.m.) (from 2 replicates). Scale bar = 100 μm. **c** Quantification of fluorescence distribution of OVA-A647 in dCLNs. *n* = 5 mice in each group. **d** Representative section images of OVA-A647-accumulated dCLNs stained with LYVE-1 and DAPI (from 2 replicates). Scale bar = 100 μm. **e** Quantification of fluorescence distribution of OVA-A647 in dCLN sections. *n* = 5 mice in each group. **f** Representative images of meninges stained with LYVE-1 and DAPI (from 2 replicates). Scale bar = 200 μm or 100 μm. **g** Quantification of area fraction of LYVE-1^+^ lymphatic vessels. *n* = 7 mice in Aged + 10 mW/cm^2^ group, *n* = 8 mice in other groups. **h** Location recognition index of novel object location (NOL) test. **i** Object recognition index of novel object recognition (NOR) test. **j** Percentage of time spent in novel arm of Y-maze test. *n* = 21 mice in Young group, *n* = 19 mice in Aged and Aged + light groups in data **h**–**j**. Data in **c**, **e**, **g**, **h**–**j** are presented as mean ± SEM, and analyzed by one-way ANOVA with Sidak’s multiple comparison test for comparisons of multiple groups. Source data are provided as a Source data file.

To determine the hypothesis that light could modulate meningeal lymphatics, a non-invasive transcranial light treatment with a laser wavelength of 808 nm was performed on aged C57BL/6J mice (15–17 months, male and female) for 4 weeks (3 times per week, 10 min per time). Mice were anesthetized, had their dorsal scalp shaved and cleaned, and then underwent transcranial illumination by 808 nm light (light-treated mice) or indoor lighting (control-treated mice) for 10 min. After the treatment, the drainage function and morphology changes of mLVs, and the mobility and cognitive function of mice were evaluated (Fig. 1a). In order to obtain an efficacious dose for light treatment, different power densities (10, 20, and 50 mW/cm^2^) were chosen for comparison, based on the conventional dose of near-infrared (NIR) light used for health benefits (5 mW/cm^2^), and the penetration rate of 808 nm light on the mouse skull with hairless scalp (about 25%, independent of output power changes) (Supplementary Fig. 1a). Additionally, the surface temperature of scalp was recorded during light irradiation. There was insignificant fluctuation of the temperature, which excluded the effect of temperature changes on meningeal lymphatic function (Supplementary Fig. 1b, c).

Firstly, drainage capacity of meningeal lymphatic system was assessed by fluorescence intensity detection of CSF tracer OVA-A647 (i.c.m.) in deep cervical lymph nodes (dCLNs). The distribution area of OVA-A647 drained into dCLNs was distinctly higher in light-treated mice than that in untreated mice (Aged +10 mW/cm^2^: 7.103 ± 1.443% versus Aged: 2.653 ± 0.76%, *P* = 0.00997; Aged + 20 mW/cm^2^: 10.338 ± 1.057% versus Aged: 2.653 ± 0.76%, *P* = 0.00006; Aged + 50 mW/cm^2^: 8.297 ± 0.748% versus Aged: 2.653 ± 0.76%, *P* = 0.00163) (Fig. 1b–e). However, there was no significant distinction of cerebral blood flow of aged mice after light treatment (Supplementary Fig. 1d, e). Structure of lymphatic vessel was evaluated by immunohistochemistry with lymphatic vessel endothelial hyaluronan receptor-1 (LYVE-1) staining (a classical marker of lymphatic endothelial cells (LECs)). The loss of area fraction of meningeal lymphatic vessels in aged mice was restored to normal by light treatment (Aged +10 mW/cm^2^: 1.605 ± 0.166% versus Aged: 1.004 ± 0.089%, *P* = 0.00112; Aged + 20 mW/cm^2^: 1.727 ± 0.111% versus Aged: 1.004 ± 0.089%, *P* = 0.00006; Aged +50 mW/cm^2^: 1.691 ± 0.063% versus Aged: 1.004 ± 0.089%, *P* = 0.00013) (Fig. 1f, g). The results demonstrated that light could improve drainage and distribution of mLVs in aged mice, and light at 20 mW/cm^2^ showed more stable and efficient effects.

To evaluate the mobility and cognitive functions of treated mice, open field (OF), novel object localization (NOL), novel object recognition (NOR) and Y-maze tests were performed on light-treated mice (20 mW/cm^2^). The results from OF test demonstrated that there was no significant difference in total distance, average velocity, or time spent in center between groups, indicating that light did not influence mobility or increase anxiety-like behavior of aged mice (Supplementary Fig. 1f–i). Interestingly, the aged mice underwent light treatment spent noticeably more time with the object at the novel location in NOL test or the novel object in NOR test than the familiar one (Aged + light: 58.92 ± 2.813% versus Aged: 41.38 ± 3.456%, *P* = 0. 00033 in NOR test), similar to the performance of young mice (1.5 months, male and female) in the same test. On the contrary, untreated aged mice preferred the familiar object in NOL and NOR test (Fig. 1h, i). Concurrently, aged mice with light treatment displayed a significantly higher discrimination index (DI, DI = (T _novel_ − T _familiar_)/(T _novel_ + T _familiar_)) in the NOL and NOR tests than untreated mice (Supplementary Fig. 1j–o). In Y-maze test, aged mice explored less in novel arm than young mice, showing deficits in memory. However, following light treatment, aged mice significantly increased the time spent in the novel arm. Interestingly, the time spent in the novel arm of light treated aged mice was similar to the performance of young mice (Fig. 1j). The above results revealed that light on mLVs could improve recognition and spatial memory in aged mice. Therefore, mLVs targeted phototherapy could effectively restore the distribution and drainage function of mLVs, as well as improve cognition in mice.

### Light modulation alleviates cognitive decline and pathological damage in AD mice

**Fig. 2:**
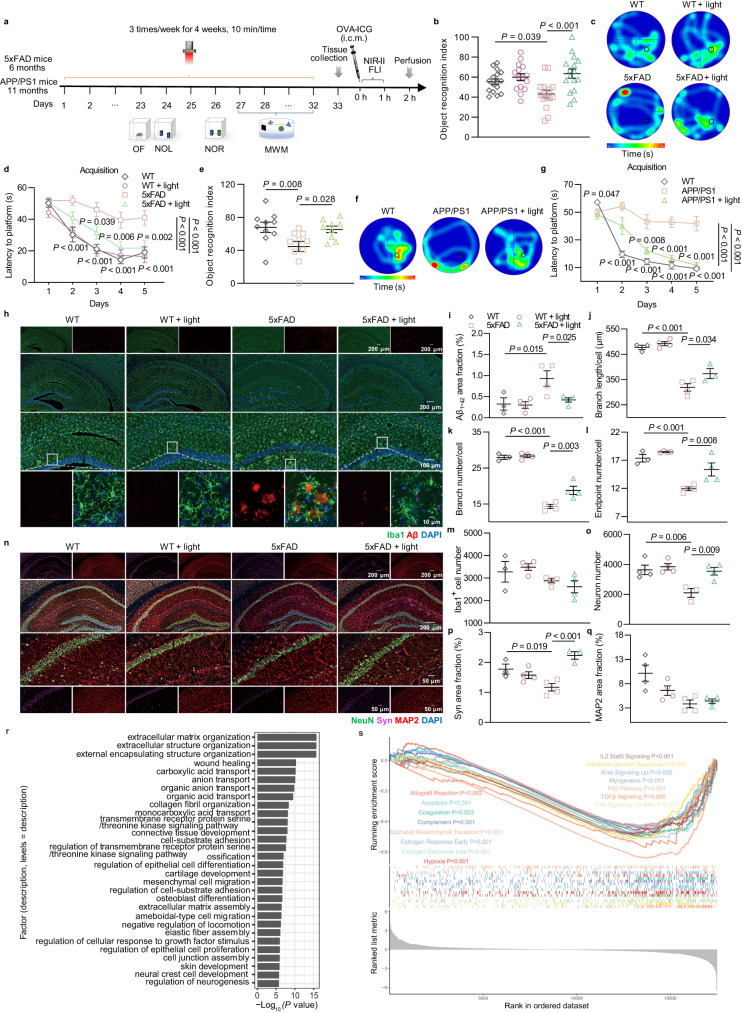
Ameliorating effects of light on cognition and pathology in AD mice. **a** Schedule of treatments and behavior tests of AD mice. **b**–**d** Object recognition index of NOR test (**b**), and representative occupancy heatmaps (**c**) and latency to platform (**d**) of MWM test of 5xFAD mice. *n* = 16 mice in WT and 5xFAD + light groups, *n* = 15 mice in WT + light and 5xFAD groups. **e**–**g** Object recognition index of NOR test (**e**), representative occupancy heatmaps (**f**) and latency to platform (**g**) of MWM test of APP/PS1 mice. *n* = 10 mice in WT and APP/PS1 groups, *n* = 8 mice in APP/PS1 + light group in data **e**; *n* = 10 mice in each group in data **g**. **h** Representative images of brain sections in hippocampus (HPC) stained with Aβ_1-42_, Iba1 and DAPI. Scale bar = 200 μm, 100 μm or 10 μm. **i** Quantification of area fraction of Aβ_1-42_ in HPC. **j**–**m** Quantification of branch length per cell (**j**), branch number per cell (**k**), endpoint number (**l**) and cell number (**m**) of Iba1^+^ cells in HPC. *n* = 3 mice in WT group, *n* = 4 mice in other groups. **n** Representative images of brain sections in HPC stained with NeuN, synaptophysin, MAP2 and DAPI. Scale bar = 200 μm or 50 μm. **o**–**q** Quantification of neuron number (**o**), and area fraction of Syn (**p**) and MAP2 (**q**) in HPC. **r** GO terms upon light treatment in 5xFAD mice. *n* = 4 mice in each group. **s** Enrichment plots of GSEA. *n* = 4 mice in each group. Data in **b**, **d**, **e**, **g**, **i**–**m**, **o**–**q** are presented as mean ± SEM, and analyzed by one-way ANOVA (**e**) or two-way ANOVA (**b**, **d**, **g**, **i**–**m**, **o**–**q**) with Sidak’s multiple comparison test for comparisons of multiple groups. *P* values in **r** and **s** were calculated with clusterProfiler using the hypergeometric test and empirical phenotype-based permutation test, respectively, without any adjustments for multiple comparisons. The statistical tests involved two-sided analysis. Source data are provided as a Source data file.

To explore the effect of mLVs modulation by light on the learning and memory ability of AD mice, we similarly conducted light treatment and performed OF, NOL, and NOR tests on 5xFAD mice (6 months, male) and APPswe/PS1ΔE9 (APP/PS1) mice (11 months, male) (Fig. 2a). In the OF test, there was no noticeable difference in total distance traveled, average speed, or time in center between groups, suggesting that mice had no motor impairments after light treatment (Supplementary Fig. 2a–d and Supplementary Fig. 3a–d). As expected, light-treated AD mice exhibited cognition restoration in the behavioral tests (5xFAD + light: 63.466 ± 4.501% versus 5xFAD: 42.987 ± 3.839%, *P* = 0.00047 in NOR test; APP/PS1 + light: 65.312 ± 3.974% versus APP/PS1: 44.732 ± 6.231%, *P* = 0.01716 in NOR test). However, there was no significant increase in cognitive performance in wild-type (WT) mice treated with light (WT + light: 59.981 ± 3.492% versus WT: 55.494 ± 2.693%, *P* = 0.63265 in NOR test) (Fig. 2b, e, Supplementary Fig. 2e–k and Supplementary Fig. 3e–g). Altogether, light also induced distinct learning and spatial memory modifications in 5xFAD and APP/PS1 mice, while not in WT mice. To further investigate the benefit of light on long-term learning and memory of AD mice, the Morris water maze (MWM) test was performed. Compared with untreated AD mice, light treatment decreased latency to find the platform in acquisition trials (5xFAD + light versus 5xFAD, *P* = 0.0000001; APP/PS1 + light versus APP/PS1, *P* < 0.0000001) and increased visits to platform location and visits to the target quadrant in probe test, without any effect on swimming velocity. However, light did not induce any behavioral indices modifications in age-matched WT mice (WT + light versus WT, *P* = 0.99929) (Fig. 2c, d, f, g, Supplementary Fig. 2l–o and Supplementary Fig. 3h–k). These results demonstrated that light could ameliorate impairments in spatial learning and memory of AD mice, but not WT mice.

Aβ accumulation, neuroinflammation and neurological damage are considered as AD pathological features. Aβ deposition and inflammation in hippocampus (HPC) and prefrontal cortex (PFC) of AD mice were evaluated by immunostaining with Aβ_1-42_ and Iba1 (Ionized calcium-binding adapter protein 1, microglial marker). The results demonstrated that aggravated brain Aβ burden occurred in HPC and PFC of 5xFAD mice, which were noticeably alleviated in light-treated 5xFAD mice to the similar level in WT littermates. Additionally, prominent microglia aggregation to Aβ and activation with reduced branch length, number and endpoints were detected in 5xFAD mice. Light diminished aberrant microglia activation in HPC displayed by increased branch length, branch number and endpoint number, with a similar tendency in the PFC. However, Iba1^+^ cell number did not change significantly but presented a decreasing trend after light treatment (Fig. 2h–m and Supplementary Fig. 4a–f). Overall, light attenuated pathological symptoms of brain Aβ deposition and neuroinflammation in AD mice.

To further examine neuroprotection of mLVs modulation by light in AD mice, immunohistochemical analyses were performed with NeuN (neuronal marker), synaptophysin (Syn) and microtubule associated protein 2 (MAP2) staining. Synaptophysin is an integral membrane protein localized to presynaptic vesicles participating in neurotransmitter transport, and MAP2 is the predominant cytoskeletal regulator within neuronal dendrites^11,12^. Compared to WT littermates, NeuN^+^ neuron cells and Syn in HPC and PFC of 5xFAD mice were significantly reduced and restored by light treatment to the similar levels as in WT littermates, indicating a significant restoration of neuronal cells. Concurrently, MAP2^+^ dendrites expression showed a slight increase in the light treatment group. Interestingly, dendrite morphology exhibited disordered distribution in HPC and PFC of 5xFAD mice, and light promoted more regular rearrangement (Fig. 2n–q and Supplementary Fig. 4g–j). Similarly, light treatment also reduced Aβ accumulation and microglia activation, as well as neuron damage and synapse loss in HPC and PFC of APP/PS1 mice (Supplementary Fig. 5a–t). Accordingly, light could elicit a neuroprotective effect via ameliorating Aβ accumulation, neuroinflammation, and neuron and synapse loss, possibly shifting CNS environment toward a mitigatory pathological stage of AD.

To further evaluate the effect of light on AD-associated pathology changes, hippocampal RNA-seq analysis of AD mice was performed. Total RNA was extracted from HPC tissue and sequenced. Volcano plot and heatmap showed expression levels of differential genes in HPC between 5xFAD mice treated with and without light (Supplementary Fig. 6a, b). Gene ontology (GO) functional enrichment analysis showed changes in gene sets involved in ion transport, cell migration, neurogenesis and development (Fig. 2r and Supplementary Fig. 6c). Kyoto Encyclopedia of Genes and Genomes (KEGG) terms enrichment also revealed transcriptional alterations related to PI3K-AKT signaling pathway, apelin signaling pathway, TGF-beta signaling pathway, oxytocin signaling pathway, and alpha-linolenic acid metabolism, which participate in neuronal survival and differentiation as well as AD progression (Supplementary Fig. 6d, e)^13–15^. In addition, Hallmark gene sets by gene set enrichment analysis (GSEA) further demonstrated distinct enrichment of down-regulated gene sets associated with AD development such as apoptosis, coagulation, hypoxia, and p53 pathway, as well as inflammation response including complement, IL2 Stat5 signaling, TNF-alpha signaling via NF-κB (Fig. 2s). These results indicated that light treatment might contribute to regulation of neuronal survival, neuroinflammation and other related pathology for AD amelioration.

### Modulation on the structure of mLVs by light enhances mLVs drainage

**Fig. 3:**
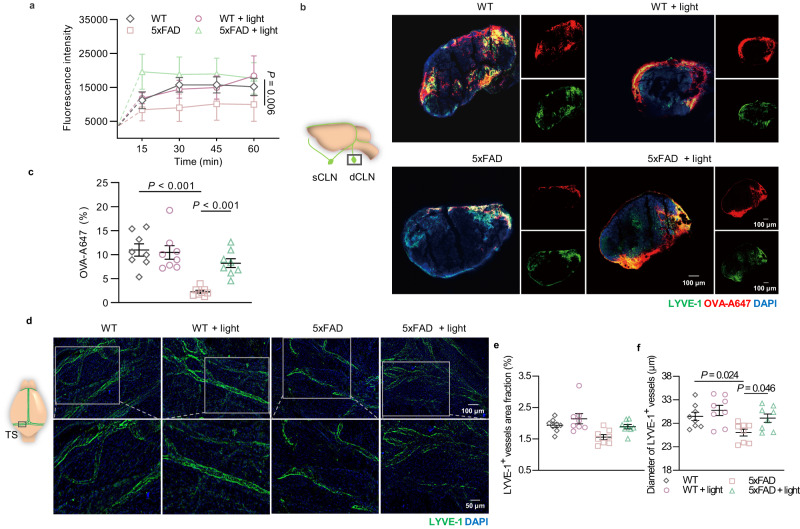
Improvement effects of light on drainage function and structural changes of mLVs in 5xFAD mice. **a** Quantification of fluorescence intensity of OVA-ICG in sCLNs after injection (i.c.m.). *n* = 8 mice in each group. **b** Representative section images of OVA-A647-accumulated dCLNs stained with LYVE-1 and DAPI (from 2 replicates). Scale bar =100 mm. **c** Quantification of fluorescence distribution of OVA-A647 in dCLN sections. *n* = 8 mice in each group. **d** Representative images of meninges stained with LYVE-1 and DAPI (from >3 replicates). Scale bar = 100 μm or 50 μm. **e**–**f** Quantification of area fraction (**e**) and diameter (**f**) of LYVE-1^+^ lymphatic vessels in meninges. *n* = 8 mice in each group. Data in **a**, **c**, **e**, **f** are presented as mean ± SEM, and analyzed by two-way ANOVA with Sidak’s multiple comparison test for comparisons of multiple groups. Source data are provided as a Source data file.

Similarly, we validated lymphatic drainage capacity of light-treated AD mice by in vivo CLNs NIR-II FLI and dCLNs immunostaining. The outflow of OVA-ICG or OVA-A647 into CLNs (5xFAD + light: 8.225 ± 0.925% versus 5xFAD: 2.249 ± 0.302%, *P* = 0.00092) or around the mLVs in meninges was higher in light-treated 5xFAD mice than that in untreated 5xFAD mice (Fig. 3a–c and Supplementary Fig. 7a–d). As expected, there was unnoticeable change of cerebral blood flow of AD mice after light treatment (Supplementary Fig. 7e, f). To investigate whether improvement of lymphatic fluid transport was associated with the structural changes of mLVs, lymphatic expansion in meninges was assessed by immunofluorescence staining with LYVE-1. The results indicated that coverage of LYVE-1^+^ vessels did not diminish in 5xFAD mice, though impaired lymphatic drainage has already been observed. However, following light treatment, increased diameter of LYVE-1^+^ vessels in meninges occurred in AD mice (5xFAD + light: 29.105 ± 0.894 μm versus 5xFAD: 26.024 ± 0.727 μm, *P* = 0.04623). Therefore, light induced structural expansion but not distribution changes of mLVs in 5xFAD mice (Fig. 3d–f). Subsequently, in APP/PS1 mice, drainage impairment and structural degeneration of mLVs were observed by diminished OVA-ICG fluorescent signals in CLNs and LYVE-1^+^ vessels area fraction in meninges, revealing degeneration of meningeal lymphatic function and distribution. Following light operation, enhancement of lymphatic drainage in AD mice was exhibited by strengthened OVA-ICG fluorescence in CLNs, as well as lymphangiogenesis by increased LYVE-1^+^ area in meninges (Supplementary Fig. 8a–d). However, there was no effects on blood vessel distribution in meninges (Supplementary Fig. 8e). As a result, light could improve drainage capacity and functional expansion of mLVs in both 5xFAD and APP/PS1 mice.

### Boosting mLVs drainage is required for cognition improvement by light

**Fig. 4:**
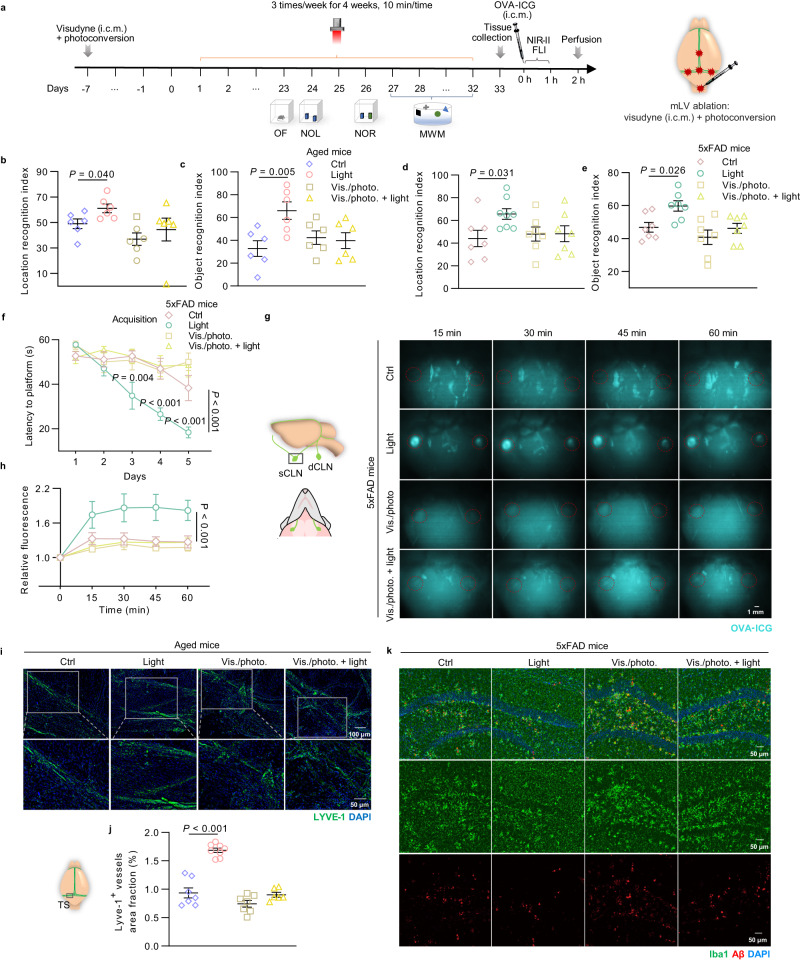
Effects of light on cognition and lymphatic drainage in mLVs-ablated mice. **a** Schedule of treatments and behavior tests of mLVs-ablated aged mice and 5xFAD mice. **b**, **c** Location recognition index of NOL test (**b**) and object recognition index of NOR test (**c**) of mLVs-ablated aged mice. *n* = 6 mice in each group. **d**–**f** Location recognition index of NOL test (**d**), object recognition index of NOR test (**e**) and latency to find platform in the acquisition of MWM test (**f**) of mLVs-ablated 5xFAD mice. *n* = 7 mice in each group in data **d**, **e**; *n* = 8 mice in Ctrl group, *n* = 9 mice in Light group, *n* = 7 mice in Vis./photo. and Vis./photo. + light groups in data **f**. **g** Representative images of sCLNs of mLVs-ablated 5xFAD mice with OVA-ICG accumulation (marked with red circles) at different time points after injection (i.c.m.) (from 2 replicates). Scale bar = 1 mm. **h** Quantification of fluorescence intensity of OVA-ICG in sCLNs. *n* = 8 mice in Ctrl, Vis./photo. and Vis./photo. + light groups, *n* = 9 mice in Light group. **i** Representative images of meninges of mLVs-ablated aged mice stained with LYVE-1 and DAPI (from 2 replicates). Scale bar = 100 μm or 50 μm. **j** Quantification of area fraction of LYVE-1^+^ lymphatic vessels. *n* = 7 mice in Ctrl and Vis./photo. groups, *n* = 8 mice in Light group, *n* = 6 mice in Vis./photo. + light group. **k** Representative images of brain sections of mLVs-ablated 5xFAD mice in HPC stained with Iba1, Aβ_1-42_ and DAPI (from 2 replicates). *n* = 4 mice in each group. Scale bar = 50 μm. Data in **b**, **c**, **d**–**f**, **h**, **j** are presented as mean ± SEM, and analyzed by two-way ANOVA with Sidak’s multiple comparison test for comparisons of multiple groups. Source data are provided as a Source data file.

To determine whether the beneficial impact of light on cognitive function and AD-associated pathology relied on the increase of mLVs drainage, we performed mLVs ablation on aged mice (15–17 months, male and female) and 5xFAD mice (6 months, male) following the procedure described in previous studies^4^. Then, we conducted light treatment (20 mW/cm^2^) on mLVs-ablated aged mice and 5xFAD mice for 4 weeks, and subsequently evaluated the motor ability, anxiety, learning and memory of mice by behavioral tests (Fig. 4a). Light had no effect on total distance, average velocity, or center time in the OF test (Supplementary Fig. 9a–d and Supplementary Fig. 10a–d), indicating no alteration in mobility or anxiety-like behavior. Similar to the results above, light treatment improved learning and memory function of aged mice and AD mice, while there were no effects in mLVs-ablated mice (Object recognition index: Vis./photo. +light of aged mice: 39.780 ± 6.939% versus Vis./photo. of aged mice: 42.339 ± 5.873%, *P* = 0.95789 in NOR test; Vis./photo. +light of 5xFAD mice: 46.170 ± 3.061% versus Vis./photo. of 5xFAD mice: 40.767 ± 4.361%, *P* = 0.47442 in NOR test) (Fig. 4b–f, Supplementary Fig. 9e–k and Supplementary Fig. 10e–n). These results showed the beneficial impact of light treatment in cognitive improvement of aged and AD mice, which was abrogated after mLVs ablation, revealing that mLVs played an important role in cognition enhancement by light treatment.

To further estimate whether abrogation of light treatment benefits in mLVs-ablated aged and AD mice is due to inability of mLVs functional modulation, drainage kinetics and mLVs morphology were evaluated. In vivo kinetic biodistribution of OVA-ICG in superficial cervical lymph nodes (sCLNs) (up to 1 h after injection), and fluorescence intensity of OVA-ICG-accumulated dCLNs and brains (at 2 h after injection) were detected and recorded by NIR-II FLI. The fluorescence intensity of sCLNs rapidly rose to peak within several minutes in light-treated aged and AD mice, then gradually declined to flatten out and maintained at a higher intensity than untreated mice. However, fluorescent signals in sCLNs of both mLVs-ablated mice with and without light treatment remained steady at lower levels within 60 min (Supplementary Fig. 9l, m and Fig. 4g, h). Finally, more OVA-ICG drained into dCLNs and brains of 5xFAD mice underwent light than untreated mice at 2 h after injection, while no difference between mLVs-ablated mice with light operation versus untreated mice (fluorescence intensity of dCLNs: 5xFAD +light: 13548.915 ± 552.890 versus 5xFAD: 9431.965 ± 568.433, *P* = 0.00006; Vis./photo. +light: 9141.019 ± 505.416 versus Vis./photo.: 9415.882 ± 741.405, *P* = 0.94270). The results showed higher fluorescence intensity of tracer in cervical lymph nodes and brains of light-treated mice, suggesting that both meningeal and glymphatic drainages were enhanced. When mLVs were ablated, in addition to the expected blockage of meningeal drainage, the beneficial impacts of light on glymphatic drainage function were also abrogated (Supplementary Fig. 10o–r). These results indicated that meningeal lymphatic and glymphatic systems constructs the flow of CSF, and light-facilitated meningeal lymphatic drainage may somehow affect the entire CSF circulatory system for macromolecule clearance. Additionally, severe loss of LYVE-1^+^ vessel area was observed in aged mice that underwent mLVs ablation, and there were no mLVs changes under light treatment (Fig. 4i, j). Similarly, the beneficial outcomes by light in regard to amelioration of microglial activation and amyloid load in HPC were also abrogated in mLVs-ablated AD mice (Fig. 4k).

### Mitochondrial homeostasis and cellular functions of meningeal lymphatic endothelial cells (mLECs) are regulated by light

**Fig. 5:**
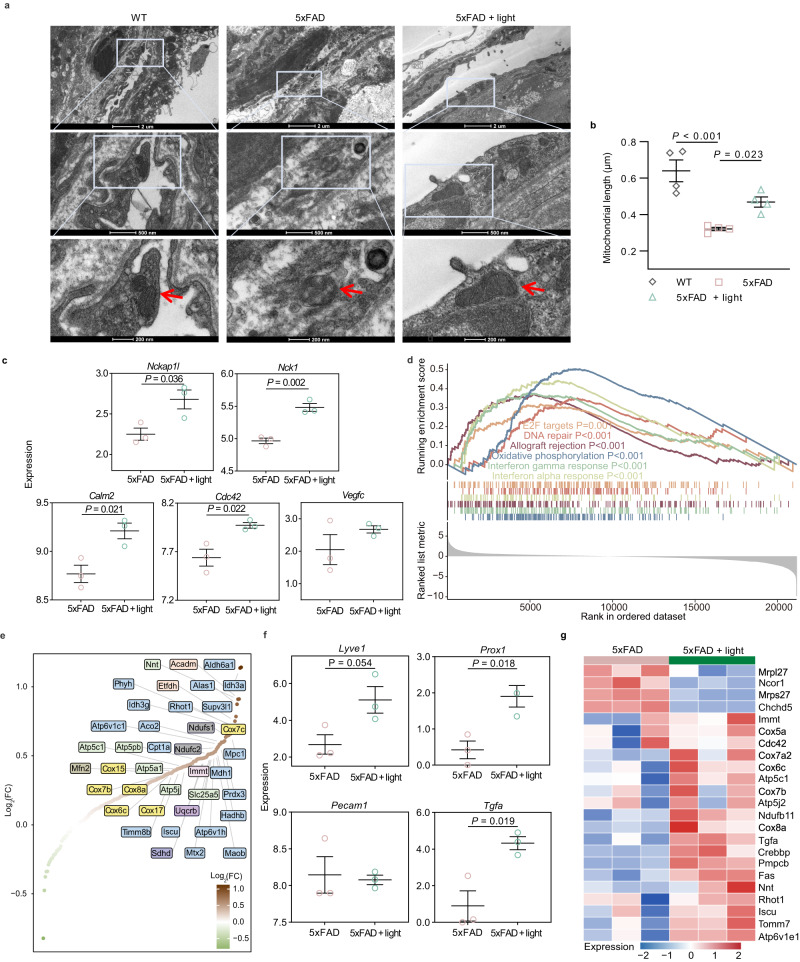
Improvement effects of light on microstructure and gene expression of mLVs in 5xFAD mice. **a** Representative transmission electron microscopy (TEM) images of mLEC arrangement and mitochondrial morphology (red arrowheads: mitochondria of mLECs) (from 2 replicates). Scale bar = 2 μm, 500 nm or 200 nm. **b** Quantification of mitochondrial length of mLECs. *n* = 4 mice in each group. **c** Expression of *Nckap1l, Nck1, Calm2, Cdc42* and *Vegfc* in meninges between light-treated AD group and AD group. **d** Enrichment plot of Hallmark gene sets by GSEA of meninges, showing the profile of the running enrichment score and positions of gene set members on the rank-ordered list. **e** Scatter plot of genes enriched in oxidative phosphorylation and ranked according to Log_2_(FC) (FC: fold change). **f** Expression of *Lyve1, Prox1, Pecam1* and *Tgfa* in mLECs between light-treated AD group and AD group. **g** Heatmap showing relative expression levels of DEGs in mLECs involved in mitochondrial metabolism. *n* = 3 mice in each group in data **c**-**g**. Data in **b**, **c**, **f** are presented as mean ± SEM, and analyzed by one-way ANOVA with Sidak’s multiple comparison test for comparisons of multiple groups (**b**), or two-tailed unpaired Student’s t-test for two-group comparisons (**c**, **f**). *P* values in **d** was calculated with clusterProfiler using the empirical phenotype-based permutation test, without any adjustments for multiple comparisons. The statistical tests involved two-sided analysis. Source data are provided as a Source data file.

To further investigate whether the beneficial effect of light on mLVs drainage relied on the functional modulation of mLECs, fine structures within mLECs of male 5xFAD mice were visualized by transmission electron microscopy (TEM). As shown in Fig. 5a, compared to age-matched WT mice, in AD mice, mLECs within mLVs were arranged more loosely, with round-shape mitochondria, as well as fractured and fuzzy mitochondrial cristae. Mitochondria are the sites of cellular energy metabolism, closely related to cellular functions. Cristae membrane and matrix of mitochondria are major functional regions of metabolism, and thus abnormal morphology of mitochondria or reduction and blurring of cristae structure directly reflect metabolism damage^16–18^. Surprisingly, in light-treated AD mice, mLECs were noticeably more orderly and compact, almost similar to WT littermates, indicating that light might rescue mLEC junction disruption. Simultaneously, light-treated AD mice showed mostly oval or rod-shaped mitochondria with much regular and clear cristae in mLECs, as well as increased mitochondrial length, revealing restored mitochondrial function by light (Fig. 5a, b). To further evaluate mitochondrial homeostasis in mLECs of light-treated mice, we measured the mitochondrial superoxide generation as well as mitochondrial membrane potential using mitochondrial superoxide indicator (MitoSOX) and tetramethylrhodamine (TMRE) probe, respectively. These results demonstrated that there was an increase in mitochondrial superoxide production and a decrease trend in mitochondrial membrane potential in mLECs of AD mice and aged mice, indicating mitochondrial dysfunction of mLECs. Light treatment rescued mitochondrial abnormalities in mLECs with less production of mitochondrial superoxide and an increased trend of mitochondrial membrane potential (Supplementary Fig. 11a–f). All in all, light promotes mitochondrial metabolism recovery of mLECs for cellular function preservation, maintaining the complete structure and compact arrangement of mLVs.

Furthermore, the cellular function of mLVs modulated by light was verified using RNA-seq analysis. Total RNA was extracted from meninge tissues and sequenced. Volcano plot and heatmap demonstrated significant difference in gene expression in meninges of 5xFAD mice treated with or without light (Supplementary Fig. 11g, h). GO enrichment of signature genes further exhibited up-regulation of cell proliferation, adhesion, differentiation, development and homeostasis, as well as transport in meninges of light-treated AD mice (Supplementary Fig. 11i). In addition, there was no distinct change of *Vegfc* expression (associated with lymphangiogenesis) in meninge after light treatment, while the genes involved in signaling of VEGF including *Nckap1l*^19^*, Nck1*^20^, *Calm2* and *Cdc42*^21,22^ significantly up-regulated (Fig. 5c). Furthermore, as shown in Supplementary Table 1, the key proteins of bicellular tight junction assembly and cell adhesion were up-regulated, suggesting that tight junction between cells was involved in the positive regulation of meningeal lymphatic vessels by light. The result demonstrated possible enhancement in cell proliferation and adhesion of mLVs after light treatment, which were the fundament for compact arrangement of mLVs. KEGG terms enriched by altered genes between groups, highlighted transcriptional changes in signaling pathway of cell development, cell communication and Alzheimer’s disease. Light treatment led to up-regulation of genes involved in cell cycle, mismatch repair and vascular smooth muscle contraction, and down-regulation of genes related to Alzheimer’s disease and pathway of neurodegeneration in meninges, which might indicate enhanced cell activity and drainage function of the meningeal drainage system, as well as AD amelioration (Supplementary Fig. 11j–l).

To further explore the mechanism of mLVs modulation, GSEA analysis was performed using the Hallmark database of gene expression. The results showed significant enrichment of 6 up-regulated gene sets associated with oxidative phosphorylation, cell cycle control, DNA repair, and immune regulation. The expression of the subset of genes contributed to oxidative phosphorylation, like *Cox7c, Cox17, Atp5pb, Mfn2, Immt* and *Iscu* as key up-regulated genes were associated with cytochrome c oxidase (CcO) activity and assembly, respiratory electron transport, adenosine triphosphate (ATP) synthesis, mitochondrial fusion, cristae formation and metabolic homeostasis (Fig. 5d, e). Oxidative phosphorylation is an energy transfer response in mitochondria, which couples with electron transport and produces ATP to provide energy for cellular synthesis and metabolism. In the process, CcO plays a key role as the terminal component of respiratory chain located in mitochondrial inner membrane, which also acts as a photoreceptor that undergoes molecular conformational change and heightens enzyme activity after light stimulation, promoting electron transfer and increasing ATP production, thus enhancing cell activity and function^23,24^. Therefore, light probably induced lymphatic expansion and led to the clustered and structured mLEC arrangement by CcO modulation for mitochondrial homeostasis and cellular function restoration, contributing to drainage dynamics recovery.

Subsequently, in order to further verify the mechanism of mLVs modulation, mLEC were isolated from meninges for RNA-seq analysis (Supplementary Fig. 12a–e). Light modulation led to up-regulated expression of *Lyve1* and *Prox1* (encoding classical markers of LECs), as well as *Tgfa* (transforming growth factor), but did not alter expression level of *Pecam1* (encoding blood vessel marker) (Fig. 5f), showing lymphangiogenesis of light-treated 5xFAD mice. In addition, up-regulated genes were also associated with oxidative phosphorylation and mitochondrial metabolism, which was consistent with meningeal RNA-seq analysis of light-treated 5xFAD mice (Fig. 5g). KEGG terms presented the pathways enriched by down-regulated genes in mLECs involved in base excision repair, mismatch repair and biosynthetic metabolism in 5xFAD mice compared with WT mice, which were up-regulated in 5xFAD mice with light modulation (Supplementary Fig. 12f, g). The results above revealed that restoration, biosynthesis and metabolism might be essential functions of mLEC for lymphatic development and transport, which were impaired in 5xFAD mice (6 months), and possibly rescued by restoration of mitochondria functions of mLECs via light modulation, further restoring the mLVs network and drainage capability for CNS homeostasis.

## Discussion

**Fig. 6:**
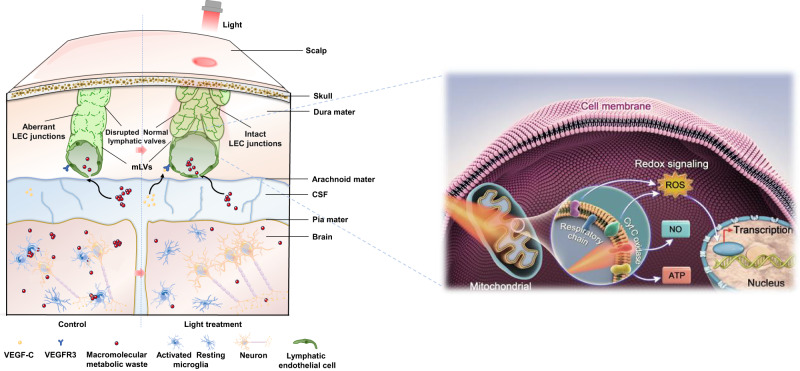
Schematic illustration of the mechanism by which mLVs-targeting phototherapy to improve AD pathology. Near-infrared light enhances mitochondrial respiratory in mLECs via photoreceptor CcO, which repairs lymphatic cell junctions and arrangement for restoration of mLVs drainage, promoting clearance of waste macromolecules (Aβ), ultimately alleviating neurodegenerative processes in mice.

In this study, we showed that non-invasive transcranial light treatment could significantly improve cognition of aged and AD mice by targeted enhancement of meningeal lymphatic drainage. We also demonstrated that light promoted mitochondrial metabolic homeostasis to improve mLEC functionality, rescuing loose junction of mLECs, which led to increase of lymphatic drainage for clearance of Aβ deposition and neuroinflammation, resulting in pathology alleviation and cognition improvement in AD mice (Fig. 6).

Meningeal lymphatic function impairment has been confirmed in various neurologic diseases, including neurodegenerative diseases, ageing, traumatic brain injury and neurotropic viral infection^4–7,9^. Here, we found that cognitive function and the capacity of lymphatic drainage impaired in 5xFAD mice (6 months), though mLVs coverage merely presented an insignificant downward trend, consistent with the finding of previous studies^5^ (Fig. 3d–f). The result suggested that damage of mLVs drainage function occurred before distributional degradation in AD mice.

Superficial-distributing mLVs in the dura mater provide a direction for transcranial neuromodulation strategy to attenuate neurologic diseases by drainage regulation. In animal cells, cytochrome c oxidase (CcO) (complex IV) was considered as the primary photoreceptor in a transparent optical window from red light (630 nm) to near-infrared light (900 nm). CcO can be boosted by photons to promote efficiency of respiratory chain and ATP synthesis for cell activity improvement and function repairation^25,26^. Particularly, 810–830 nm has been found to have the deepest penetration with limited absorption either by water or blood in tissues, which is more suitable for transcranial modulation for long-term pathological improvement.

Previous studies confirmed that transcranial light treatment at 1267 nm could enhance the clearance of injected Aβ peptide from the brain, shown by accumulated Aβ in the brain and dCLN^27,28^. Recently, transcranial light treatment at 1267 nm was performed in male newborn and adult rodents for treatment of intraventricular hemorrhage by enhancing the removal of red blood cells through brain lymphatics^29^. Nevertheless, how 1267 nm light regulates lymphatic transport remains unclear. It is critical to determine the regulatory mechanism of meningeal lymphatic drainage by light and how drainage changes participate in cognitive improvement. By further observing the fine structure of mLECs with TEM imaging, we found changes in mitochondrial morphology with mostly oval or rod shape and regular cristae distribution in mLECs of light-treated AD mice, as well as increased mitochondrial length, implying mitochondrial function recovery for cellular function enhancement (Fig. 5a, b). Moreover, meningeal RNA-seq analysis showed positive regulation of oxidative phosphorylation and metabolic homeostasis for enhancement of cell growth, probably further promoting *Cdc42*^22^ and *Ocln* expression for cell migration and restoration of endothelial adherens junctions in 5xFAD mice with light (Supplementary Fig. 11c, Fig. 5c, d and Supplementary Table 1). Additionally, the up-regulated genes contributed to oxidative phosphorylation associated with CcO activity, respiratory electron transport, adenosine triphosphate (ATP) synthesis and mitochondrial metabolic homeostasis (Fig. 5e). Furthermore, mLEC RNA-seq analysis demonstrated the up-regulated expression of oxidative phosphorylation-, metabolism- and lymphangiogenesis-associated genes and confirmed restoration of mitochondrial metabolism and cellular development of mLECs in light-treated 5xFAD mice (Fig. 5f, g and Supplementary Fig. 12f, g). These results indicated that light stimulation on CcO is able to enhance oxidative phosphorylation of mitochondria, promoting energy availability and signal transduction for cellular function maintenance of mLECs, further mediating repair of endothelial junctions. The modulation of mLEC function by light enhances lymphatic transport capacity for clearance of waste macromolecules, further alleviating AD-associated pathological injury by reducing Aβ deposition, neuroinflammation and neuronal damage, finally contributing to cognitive improvement.

For the beneficial outcomes by NIR light treatment, it is possible that other regulatory mechanisms may coexist simultaneously. Light may increase the activity of the transporting immune cells in dura, thereby regulating the meningeal immune system for cerebral homeostasis. ln addition, direct regulation of neuronal activity on the cerebral cortex may be another possible mechanism by which light regulates brain function for cognition improvement. Interestingly, light was ineffective in regulation of cognition in WT mice, probably owing to the ceiling effect of existing draining ability^4^ (Fig. 3a–c and Supplementary Fig. 7a–d). Light may play a role in the prevention or amelioration of cognitive deficits depending on the time of treatment. In the future, we plan to investigate whether maintaining lymphatic drainage function by periodic light treatment at the early disease stages in AD animal models could alleviate or delay disease progression.

In our study, we confirmed that mLVs-targeting phototherapy enhanced cognition in aged and AD mice. Furthermore, we revealed that light could restore mitochondrial metabolism and cellular function of mLECs, which contribute to cell arrangement amelioration of mLVs to improve drainage dynamics for pathology relief in AD mice. Overall, we provide a potential treatment for neurologic diseases by mLVs transport modulation for CNS waste clearance, which could alleviate cognitive impairment and related pathology.

## Methods

### Animals

All the mice were in C57BL/6J background purchased from Jinzhihe Biotechnology Co., Ltd (Yangzhou, China). Male and female WT mice aged between 15 to 17 months served as the elderly model, and 1.5-month-old ones served as the counterpart young group. Two male AD models were used in this study: 6-month-old 5xFAD and 11-month-old APPswe/PS1ΔE9 (APP/PS1) mice, with their transgene non-carrier littermates as counterpart groups. Amyloid deposition accompanied by gliosis is seen in 5xFAD mice at 2 months of age. Cognitive deficit occurs at about 5 months old and neuron loss in the brain regions with amyloidosis begins at 6 months of age^30^. In APP/PS1 mice, amyloid deposits in brain between 4 to 6 months and cognitive deficit occurs between 6 to 10 months^31,32^. Mice of all strains were bred under 12-h light and darkness alternating cycles with controlled temperature (20–23 °C) and humidity (50–60%), and were allowed free access to standard diet and water. The mice treatments were performed in compliance with the Guide for the Care and Use of Laboratory Animals. All animal studies were reviewed and approved by the Institutional Animal Care and Use Committee of Hainan University (approval number: HNUAUCC-2021-00025).

### Intra-cisterna magna (i.c.m.) injections

Mice were anesthetized with 1% isoflurane supplemented with dexmedetomidine. The dorsal surface of neck was shaved and cleaned. After making a 1.5 cm incision of the neck skin at midline, the muscle layers were retracted with forceps for the cisterna magna exposure. The desired solution was injected into the cisterna magna compartment (2.5 μl/min). For evaluation of lymphatic drainage, 10 μl of Alexa Fluor 647 conjugate ovalbumin (at 0.5 mg/ml, OVA-A647, Thermo Fisher Scientific, USA) or OVA-ICG (at 12.5 μg/ml) was administered using a Hamilton syringe. After the injection, the needle was held in place for an extra 2 min to avoid CSF backflow. Finally, the neck skin and muscle were cleaned and sutured, and the mice were placed on a warming pad for recovery from anesthesia until conscious, then transported back to home cage.

### Meningeal lymphatic vessels (mLVs) ablation

Visudyne treatment was performed as published protocols^4^. Mice were anesthetized with 1% isoflurane supplemented with dexmedetomidine. Then 10 μl visudyne (at 0.5 mg/ml, APExBIO, USA) was injected (i.c.m.) at a rate of 2.5 μl/min. After 15 min, visudyne was photoconverted with a non-thermal 689 nm light (LSR689CP-3.6W-FC, LASEVER, China) on five different spots on skull (one at the injection site, one at the confluence of the sinuses, two at the bilateral transverse sinuses and one at the superior sagittal sinus), with 50 J/cm^2^ irradiation (600 mW/cm^2^, 83 s each spot). The scalp skin was sutured and the mice were placed on a warming pad for recovery from anesthesia until conscious, then transported back to home cage.

### Light penetration measurement

Mice were euthanized by cervical dislocation and the scalps were shaved and cleaned with razor and depilatory cream. Then the head was isolated, the lower jaw and brain tissue was removed, and the skull with hairless scalp was harvested. The skull was fixed under the 808 nm light source. The penetrations of the skull with hairless scalp at lambda point under the light at different powers were recorded (powers detected by a Digital Handheld Optical Power and Energy Meter (PM100D, Thorlabs Inc, Newton, NJ, USA)). The transmittances were calculated as radiation flux/incident power.

### Light treatment

Mice were brought into experimental room at least 30 min before light treatment for habituation. Mice were anesthetized with 1–1.5% isoflurane, and dorsal scalp was shaved and cleaned. Then a continuous 808 nm light (LSR808H-5W-FC, LASEVER, China) was applied to illuminate the skull with intact hairless scalp over an area of 0.8 cm^2^, at 10–50 mW/cm^2^ for 10 min, which ended up with doses of 6–30 J/cm^2^. The surface temperature of scalp was measured along the 10 min of exposure to the light irradiation using a near-infrared (NIR) thermal camera (226S, FOTRIC, China) and analyzed using AnalyzIR (version 4.3.2.71). Mice underwent light treatment three times a week lasting for continuous four weeks. For the mice in control group, the same experimental procedure was performed. Mice were anesthetized with isoflurane, and dorsal scalp was shaved and cleaned, then underwent transcranial illumination by indoor lighting for 10 min. After treatments, the mice were placed on a warming pad for recovery from anesthesia until conscious, then transported back to home cage.

### Cervical lymph nodes (CLNs) imaging

Mice were anesthetized with 1% isoflurane supplemented with dexmedetomidine, and neck skin was resected to expose CLNs. The dorsal surface of neck was cleaned and incised. The muscle layers were retracted to both sides to expose the cisterna magna. Then 10 μl of OVA-ICG (at 12.5 μg/ml) was administered (i.c.m.) to mice at 2.5 μl/min using a Hamilton syringe. After the injection, the needle was held for additional 2 min to avoid CSF backflow. Finally, the neck skin and muscle were cleaned and sutured, and the mice were allowed to recover on a warming pad with 1% isoflurane maintenance. Fifteen minutes post-injection start, the mice were placed supine under the MARS in vivo imaging system (Artemis Intelligent Imaging, China) for imaging. Images of CLNs were collected 15 min to 1 h after injection start, with intervals of 15 min. The time-dependent fluorescence intensity of accumulated tracers in CLNs was recorded. Two hours after injection start, the mice were transcardially perfused with PBS and 4% paraformaldehyde (PFA). Cervical muscles were cut and retracted, and sCLNs and dCLNs were collected to be imaged by the imaging system. Quantitative analysis of fluorescence intensity was performed using Fiji software (version 1.53q) and LightField (version 6.14) to characterize the lymphatic drainage function.

### Laser speckle contrast imaging (LSCI)

After one-month treatment, mice were anesthetized with 1% isoflurane supplemented with dexmedetomidine, followed by the scalp and the fascia resected to expose the skull. Then the mice were head-fixed using a head-fixation frame and placed prostrate under the LSCI microscope (Simopto, China) for detection of cerebral blood flow. The signals excited by 785 nm laser were amplified and digitized for blood flow imaging. The relative blood flow (%) of mice was record for continuous 3 min and the blood flow index (perfusion units, PU) was calculated by SIM BFI software (version 3.1.45, Simopto).

### Open field (OF) test

OF test was performed in an opaque cubic arena (40 cm edge). Before the test, mice were habituated to the test room for 30 min. Mice were then placed into the arena and allowed to explore for 10 min. Total distance (cm), velocity (cm/s) and time spent in the center (%) were recorded and analyzed using DigBehv behavior analysis system (version 4.1, Jiliang Software Science&Technology Co., Ltd.).

### Novel object recognition/novel object location (NOR/NOL) test

NOR or NOL task was conducted in the same cubic arena used in the OF test. 24 h before the test, the mice were placed in the arena to explore for 10 min as habituation. Then two identical objects were placed in different corners, around 6 cm away from the walls. During training, mice were allowed to explore the objects in the arena for 10 min and then return to the home cages. 1 or 24 h later, memory of the location or object recognition was tested. For NOL test, mice were allowed to explore the objects for 10 min in the same arena with one object moved to a novel location. For NOR test, mice were allowed to explore for 10 min in the same arena with one object replaced with a novel one in the original position. Object exploration was recorded using Visutrack software (version 3.0, Xinruan Information Technology Co., Ltd.) and recognition index (RI), discrimination index (DI) and object preference (percentage of time with object) were calculated. RI = T _novel_/(T _novel_ + T _familiar_), DI = (T _novel_ − T _familiar_)/(T _novel_ + T _familiar_), object preference = T _novel_/(T _novel_ + T _familiar_) or T _familiar_/(T _novel_ + T _familiar_).

### Y-maze test

Y-maze test was conducted in a Y-shaped maze with three identical arms, each 35 cm length, 6 cm width and 15 cm height. Different geometrical figures were placed inside each arm as spatial landmarks. The arms were randomly designated as the novel, start and other arm. During training, a divider was used to block the novel arm. The mice were placed into the start arm of the maze, heading away from the maze center, for a 10-min exploration. In test trial, the mice were placed into the Y-maze 1 h later to explore for 5 min with all arms open to free access. The time spent in each arm was recorded using DigBehv behavior analysis system (version 4.1, Jiliang Software Science&Technology Co., Ltd.) and percentage of time spent in the novel arm (%) was calculated.

### Morris Water Maze (MWM) test

MWM test was conducted in a circular pool (120 cm diameter) filled with opaque water at around 23 °C to a 30 cm depth. Different geometrical figures were placed surrounding the pool as spatial reference landmarks. During five continuous training days, mice were placed into the tank to find a hidden fixed platform (10 cm diameter, located at 1 cm below the water surface) within 60 s. The mice failing to find the platform during 60 s were gently guided to climb on the platform and rest for 15 s. The latency to platform was recorded using Visutrack software (version 3.0, Xinruan Information Technology Co., Ltd.) and the mean latency to platform of the four trials each day was calculated. On the sixth day (probe trial), the platform was removed and mice performed a 60-s exploration. The percentage of time spent in each quadrant (%), the number of crossings of the platform area and swimming velocity (cm/s) were recorded and analyzed.

### Tissue collection

Mice were anesthetized and then transcardially perfused with PBS and 4% PFA. Cervical skin and muscles were cut and retracted, and CLNs were collected and fixed in 4% PFA for 24 h at 4 °C. The skin and muscle of the heads were removed from the bones, and then brains and skulls were collected and fixed in 4% PFA for 24 h at 4 °C. Then the whole-mount meninges were carefully stripped from skulls and then stored in PBS at 4 °C until further use. The fixed brains and CLNs were washed with PBS, dehydrated with 10%, 20%, and 30% sucrose for three days and frozen in Tissue-Tek OCT compound (SAKURA, USA) at −80 °C. The brains were sliced (50-μm sections) and kept in cryoprotectant at −20 °C for further use.

### Immunohistochemistry and imaging

For antigen retrieval, the frozen brain or CLN sections were heated with citrate buffer for 30 min. For immunofluorescence staining, brain sections, CLN sections and meninges were washed with PBS and blocked with PBS containing 0.3% Triton X-100 (Sigma, USA) and 5% bovine serum albumin (BSA, Sigma) for 1 h at room temperature (RT), followed by incubation with of primary antibodies dilutions overnight at 4 °C: rat anti-LYVE-1 (cat# 14-0443-82, lot# 2494966, clone# ALY7, 1:200, Invitrogen, USA), mouse anti-amyloid-β_1-42_ (cat# 805501, lot# B326654, clone# 12F4, 1:1000, Biolegend, USA), rabbit anti-Iba1 (cat# 019-19741, lot# LEN4341, 1:500, Abcam, UK), rat anti-NeuN (cat# ab279297, lot# GR3400636-6, clone# EPR12763, 1:1000, Abcam, UK), rabbit anti-Syn (cat# MA5-14532, lot# WI3369463, clone# SP11, 1:100, Invitrogen), mouse anti-MAP2 (cat# MA5-12826, lot# XF3603732A, clone# AP18, 1:200, Invitrogen), rabbit anti-CD31 (cat# SAB4502167-100UG, lot# 210547, 1:200, Sigma). Tissues were then washed three times with PBS, followed by incubation with Alexa Fluor 488/555/633 goat anti-rat/rabbit/mouse IgG (H + L) secondary antibodies in PBS containing 0.3% Triton X-100 for 1 h in dark at RT: Alexa Fluor 488 goat anti-rat IgG (H + L) secondary antibody (cat# A-11006, lot# 2247986, 1:500, Invitrogen), Alexa Fluor 488 goat anti-rabbit IgG (H + L) secondary antibody (cat# A-32731, lot# VG302077, 1:500, Invitrogen), Alexa Fluor 555 goat anti-rat IgG (H + L) secondary antibody (cat# A-21434, lot# 2184321, 1:500, Invitrogen), Alexa Fluor 555 goat anti-rabbit IgG (H + L) secondary antibody (cat# A-32732, lot# YA361054, 1:500, Invitrogen), and Alexa Fluor 633 goat anti-mouse IgG (H + L) secondary antibody (cat# A-21052, lot# 2418505, 1:500, Invitrogen). Then tissues were washed three times and incubated with DAPI for 5 min at RT. Finally, tissues were mounted with SlowFade Diamond Antifade Mountant (Invitrogen) and imaged by confocal laser scanning microscopy (FV3000, Olympus, Japan) or Slide Scanner (VS200, Olympus). Image export and quantitative analysis were performed using OLYMPUS OlyVIA (version 4.1) and Fiji software (version 1.53q).

### Mitochondrial superoxide and mitochondrial membrane potential measurement

MitoSOX Red (Invitrogen) and tetramethylrhodamine (TMRE, Invitrogen) probe were used to assess the mitochondrial superoxide and mitochondrial membrane potential levels in meningeal lymphatic endothelial cells (mLECs). The fresh whole-mount meninges were collected and incubated in MitoSOX reagent for 10 min or in TMRE reagent for 30 min at 37 °C, followed by Alexa Fluor 488 rat anti-LYVE-1 (cat# 53-0443-80, lot# 2547846, clone# ALY7, 1:250, Invitrogen) dilution incubation for 30 min at 37 °C. MLECs were imaged by a confocal microscope (FV3000, Olympus).

### Transmission electron microscopy (TEM)

Mice were euthanized by cervical dislocation, then the skullcaps were dissected and the whole-mount meninges were stripped. MLVs regions of meninges were trimmed to 5 mm × 1 mm tissues with a blade and rinsed twice with PBS. Then tissues were fixed with 2% glutaraldehyde buffer with 2% paraformaldehyde for 30 min at RT and stored at 4 °C until processed as TEM samples. Then the tissues were rinsed with PBS, secondly fixed with 1% osmium tetroxide (OsO4), rinsed with double-distilled water, dehydrated with ethanol, infiltrated with acetone, and embedded in epoxy resin. The samples were cut as 70 nm sections, stained with uranyl acetate and lead citrate, and then imaged using a transmission electron microscope (Tecnai G2 Spirit, FEI, USA). Quantitative analysis of the mitochondrial length of meningeal lymphatic endothelial cells (mLECs) was performed using Fiji. The images of LECs in the meninges were acquired using a transmission electron microscopy and the mean of 10 individual mitochondrial length measurements was calculated for each sample in different groups (*n* = 4).

### Sorting of mLECs

To obtain a suspension of mLECs of 5xFAD mice using fluorescence-activated cell sorting (FACS), mice were anesthetized and then transcardially perfused with PBS. Skullcaps were dissected and whole-mount meninges were stripped in Dulbecco’s Modified Eagle Medium (DMEM, Gibco, USA), supplied with 10% FBS (Gibco), penicillin (50 U/ml, Gibco) and streptomycin (50 mg/mL, Sigma). Then meninges were incubated in DMEM containing 1 mg/ml of collagenase VIII (Sigma) and 35 U/mL of DNase I (Sigma) for 30 min at 37 °C. The digested meninges were filtrated through 70-μm nylon cell meshed for single-cell suspensions. Cells were then centrifuged (280 g) at 4 °C for 10 min and resuspended in PBS. Then cells were incubated with antibodies dilutions at 4 °C for 30 min: rat anti-CD45-PE (cat# 12-0451-82, lot# 2356222, clone# 30-F11, 1:150, eBioscience), rat anti-CD31-FITC (cat# 11-0311-85, lot# 2373735, clone# 390, 1:100, eBioscience), hamster anti-podoplanin-PE-Cy7 (cat# 127412, lot# B355692, clone# 8.1.1, 1:100, Biolegend) and rat anti-CD11b-PerCP (cat# 101230, lot# B352939, clone# M1/70, 1:80, BioLegend), and finally incubated with DAPI for 5 min. Cells were then resuspended in FACS buffer. The singlets were gated using the pulse width of the side scatter and forward scatter. Cells negative for DAPI were identified and selected as viable cells. The single DAPI^−^CD45^−^CD31^+^PDPN^+^ cells were gated and sorted using flow cytometry (MoFlo Astrios, Beckman Coulter, USA). The sample cells were dispensed in the droplets with a volume of 1 μl and dropped into the PCR tubes containing 4 ul of lysis buffer. The samples were temporarily stored at −80 °C for further analysis.

### RNA extraction and sequencing

All RNA sample processing (including cDNA library generation) and RNA sequencing (RNA-seq) were performed by E-GENE Technology Co., Ltd (Shenzhen, China). The RNA-seq libraries for samples were prepared using the Smart-seq2 protocol^33^ with minor modifications. For tissue samples, the total RNA of each simple was extracted from one individual hippocampus (HPC) tissue of brain (*n* = 4) or one individual meninge tissue (*n* = 3) via TRIzol total RNA extraction reagent (Thermo Fisher Scientific). Then RNA of sample was quantified by Qubit Fluorometer and RNA integrity was detected by agarose gel electrophoresis. For LEC samples, the LECs (previously sorted by FACS from one individual meninge, *n* = 3) were lysed with 4 μl of 0.2% Triton X-100 solution containing 0.5 μl of RNase inhibitor. The extracted total RNA or the cell lysis was mixed with 1 μl of 10 μM Oligo-dT primer for RNA enrichment, and was subjected to reverse transcription in 1X Super Script II first-strand buffer system with 200 U of Super Script II reverse transcriptase, 0.1 μl of Template switch oligo (TSO, 100 μM), 20 U RNase Inhibitor, 0.75 μl of Dithiothreitol (DTT, 100 mM), 3 μl of Betaine (5 M) and 0.09 μl of MgCl_2_ (1 M). The full-length cDNA was synthesized and amplified with 10 to15 cycles of PCR amplification. Then the product was purified and used for library construction. After quantified by Agilent 2100 Bioanalyzer (Agilent Technologies, USA), the library was sequenced with Illumina Novaseq platform.

The raw reads were filtered by fastp^34^ (version 0.18.0) to remove adapters or low-quality bases for following assembly and analysis. Fragment per kilobase of transcript per million mapped reads (FPKM) value was calculated to quantify expression abundance and variations of transcription using RSEM^35^ software. All analyses of visualizing the RNA-seq data were performed with the statistical software R (version 4.3.1). Differential expression analysis was conducted and visualized using the R package DESeq2^36^ (version 1.40.2) and ggplot2 (version 3.4.2). The genes with a fold change value greater than 2.0 or lower than 0.5 and false discovery rate (FDR)-corrected *P* < 0.05 were considered significantly differentially expressed genes (DEGs). Gene Ontology (GO) and Kyoto Encyclopedia of Genes and Genomes (KEGG) pathway enrichment analysis provided GO and KEGG terms that significantly enriched in DEGs comparing to the genome background, and filtered the DEGs that correspond to biological functions or pathways. GO and KEGG term enrichment analysis were carried out with the clusterProfiler software (version 4.8.3). GO terms similarity based on similarity matrices of functional terms was performed using R packages simplifyEnrichment (version 1.10.0). In order to gain a better understanding of the mechanism, a Gene Set Enrichment Analysis (GSEA) was performed by ranking all genes based on their level of difference using R package clusterProfiler (version 4.8.3) and visualized using R package enrichplot (version 1.20.3). The hallmark gene sets served as reference sets for GSEA were obtained from the Molecular Signatures Database (https://www.gsea-msigdb.org/gsea/msigdb). Heatmap were visualized using ggplot2 (version 3.4.2) and pheatmap (version 1.0.12). We didn’t make any adjustments on data, and the data were sequenced on the same platform and processed in the same way.

### Statistical analysis

To ensure randomization, animals from different cages were divided in the same experimental group. Experimenters were blind to the group allocation of mice in all the experiments during data collection and analysis. Statistical significance was conducted using two-tailed unpaired Student’s *t* test for two-group comparisons, and one-way ANOVA or two-way ANOVA with Sidak’s multiple comparison test for multiple-group comparisons. A *P* value less than 0.05 was considered statistically significant. Data are presented as mean ± SEM. Statistical data were analyzed using GraphPad Prism (version 8.4.2, GraphPad Software, Inc.).

### Reporting summary

Further information on research design is available in the Nature Portfolio Reporting Summary linked to this article.

### Supplementary information

Supplementary information

Reporting Summary

### Source data

Source data

## Data Availability

The data that support the findings of this study are available within the article, the Supplementary Information files and the Source Data files. The RNA sequencing data generated for this study can be found in the GEO repository under accession number GSE245658. The hallmark gene sets served as reference sets for GSEA in RNA-seq analysis were obtained from the Molecular Signatures Database (https://www.gsea-msigdb.org/gsea/msigdb/). Source data are provided with this paper.

## Code availability

Customized code or algorithms were not used to generate results in the present study.
