# Supplementary material for: Non-invasive modulation of meningeal lymphatics ameliorates ageing and Alzheimer’s disease-associated pathology and cognition in mice
Source: Nat Commun. 2024 Feb 16;15:1453. doi: 10.1038/s41467-024-45656-7 (PMC10873306; doi:10.1038/s41467-024-45656-7)
Supplement: Supplementary file 1 — Supplementary information [file 41467_2024_45656_MOESM1_ESM.pdf]

Supplementary Information

Title: Non-invasive modulation of meningeal lymphatics ameliorates ageing and Alzheimer's disease-associated pathology and cognition in mice

Authors: Miao Wang[1], Congcong Yan[1], Xi Li[1], Tianhao Yang[1], Shengnan Wu[2], Qian Liu[1], Qingming Luo[1]*, Feifan Zhou[1]*

Affiliations:

1. State Key Laboratory of Digital Medical Engineering, School of Biomedical Engineering, Hainan University, Haikou 570100, China
2. Key Laboratory of Brain Health Intelligent Evaluation and Intervention, Ministry of Education, School of Medical Technology, Beijing Institute of Technology, Beijing 100081, China

Corresponding author: Feifan Zhou, zhouff@hainanu.edu.cn; Qingming Luo, qluo@hainanu.edu.cn

This file contains the following Supplementary Figures and Tables:

Supplementary Fig. 1-Effects of light on mobility and cognition in aged mice.

Supplementary Fig. 2-Effects of light on mobility and cognition in 5xFAD mice.

Supplementary Fig. 3-Effects of light on mobility and cognition in APP/PS1 mice.

Supplementary Fig. 4-Ameliorating effects of light on AD-associated pathology in prefrontal cortex (PFC) of 5xFAD mice.

Supplementary Fig. 5-Ameliorating effects of light on AD-associated pathology in HPC and PFC of APP/PS1 mice.

Supplementary Fig. 6-Improvement effects of light on gene expression of HPC in 5xFAD mice.

Supplementary Fig. 7-Improvement effects of light on drainage function changes of mLVs in 5xFAD mice.

Supplementary Fig. 8-Improvement effects of light on drainage function and structural changes of mLVs in APP/PS1 mice.

Supplementary Fig. 9-Effects of light on cognition and lymphatic drainage in mLV-ablated aged mice.

Supplementary Fig. 10-Effects of light on cognition and lymphatic drainage function in mLV-ablated 5xFAD mice.

Supplementary Fig. 11-Improvement effects of light on gene expression of meninges in 5xFAD mice.

Supplementary Fig. 12-Improvement effects of light on gene expression of mLECs in 5xFAD mice.

Supplementary Table 1-Expression of genes involved in tight junction and cell adhesion in meninges between light-treated AD group versus AD group.

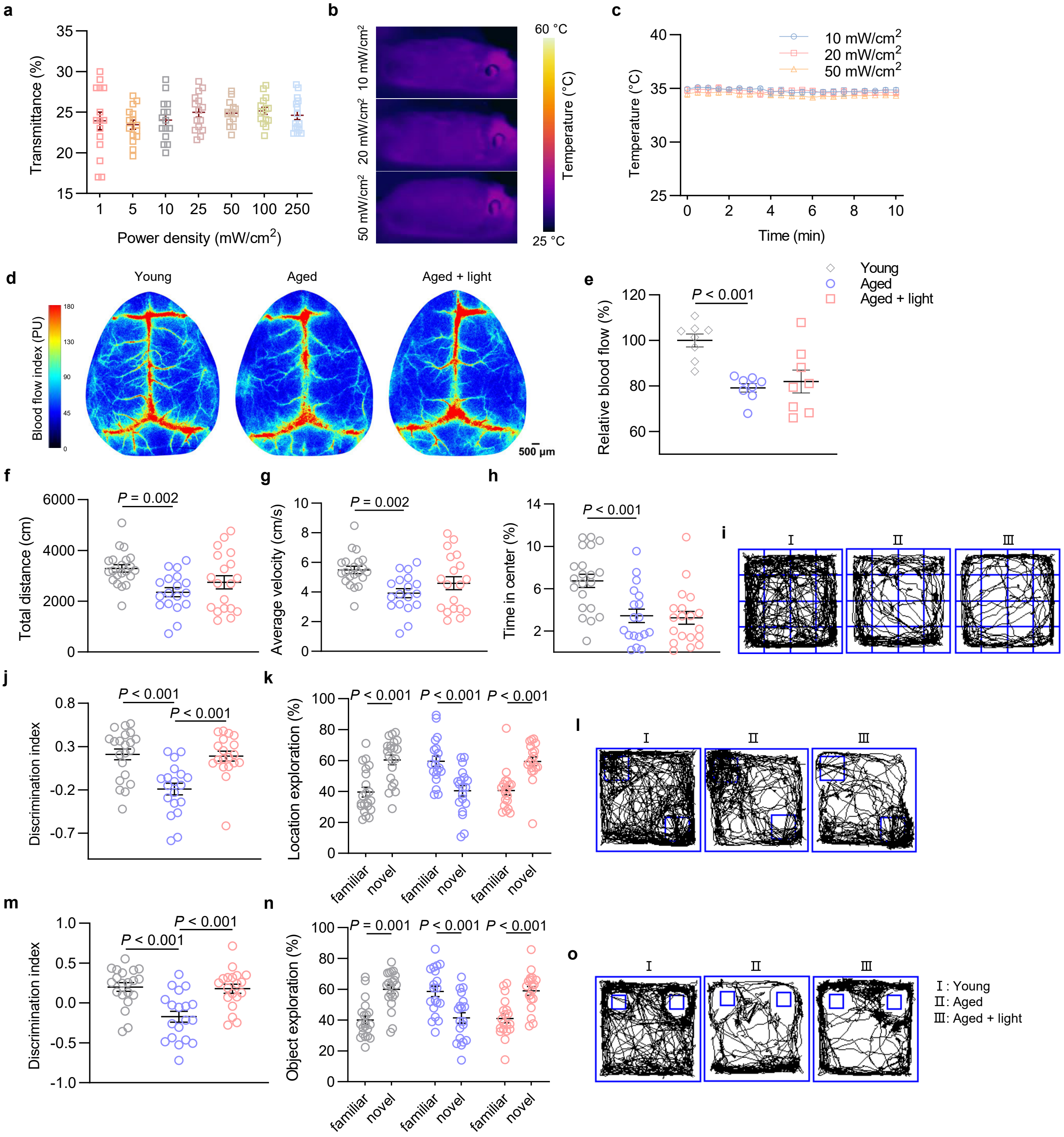

a
Transmittance (%)
Power density (mW/cm2)
b
10 mW/cm2
20 mW/cm2
50 mW/cm2
60 °C
25 °C
Temperature (°C)
c
Temperature (°C)
Time (min)
10 mW/cm2
20 mW/cm2
50 mW/cm2
d
Young
Aged
Aged + light
Blood flow index (PU)
500 μm
e
Relative blood flow (%)
P < 0.001
Young
Aged
Aged + light
f
Total distance (cm)
P = 0.002
g
Average velocity (cm/s)
P = 0.002
h
Time in center (%)
P < 0.001
i
Ⅰ
Ⅱ
Ⅲ
j
Discrimination index
P < 0.001
P < 0.001
k
Location exploration (%)
P < 0.001
P < 0.001
P < 0.001
familiar
novel
l
m
Discrimination index
P < 0.001
P < 0.001
n
Object exploration (%)
P = 0.001
P < 0.001
P < 0.001
familiar
novel
o
Ⅰ: Young
Ⅱ: Aged
Ⅲ: Aged + light

Supplementary Fig. 1 | Effects of light on mobility and cognition in aged mice. a Transmittance of mouse skull with hairless scalp under 808 nm light. n = 15 mice in each group. **b-c** Representative IR thermal images (**b**) and temperature curves (**c**) of mouse scalps under 808 nm light irradiation at 10, 20 and 50 mW/cm^2. n = 4 mice in 10 mW/cm^2 and 20 mW/cm^2 groups. n = 5 mice in 50 mW/cm^2 group. **d** Representative images of blood flow obtained by laser speckle contrast imaging (LSCI) of brain/meningeal vasculature of aged mice (from 2 replicates). Perfusion units, PU. **e** Relative blood flow of aged mice after the treatments. n = 8 mice in each group. **f-i** Total distance (**f**), average velocity (**g**), time spent in center (**h**) and representative test paths (**i**) of open field (OF) test. n = 21 mice in Young group; n = 18 mice in Aged group; n = 19 mice in Aged + light group in data **f-h**. **j-l** Discrimination index (**j**), percentage of object exploration (**k**) and representative test paths (**l**) of novel object location (NOL) test. **m-o** Discrimination index (**m**), percentage of object exploration (**n**) and representative test paths (**o**) of novel object recognition (NOR) test. n = 21 mice in Young group; n = 19 mice in Aged and Aged + light groups in data **j**, **k**, **m**, **n**. Data in **a**, **c**, **e**, **f-h**, **j**, **k**, **m**, **n** are presented as mean ± SEM, and analyzed by one-way ANOVA (**a**, **e**, **f-h**, **j**, **m**) or two-way ANOVA with Sidak's multiple comparison test for comparisons of multiple groups (**c**, **k**, **n**). All the measurements were taken from distinct samples. Source data are provided as a Source data file.

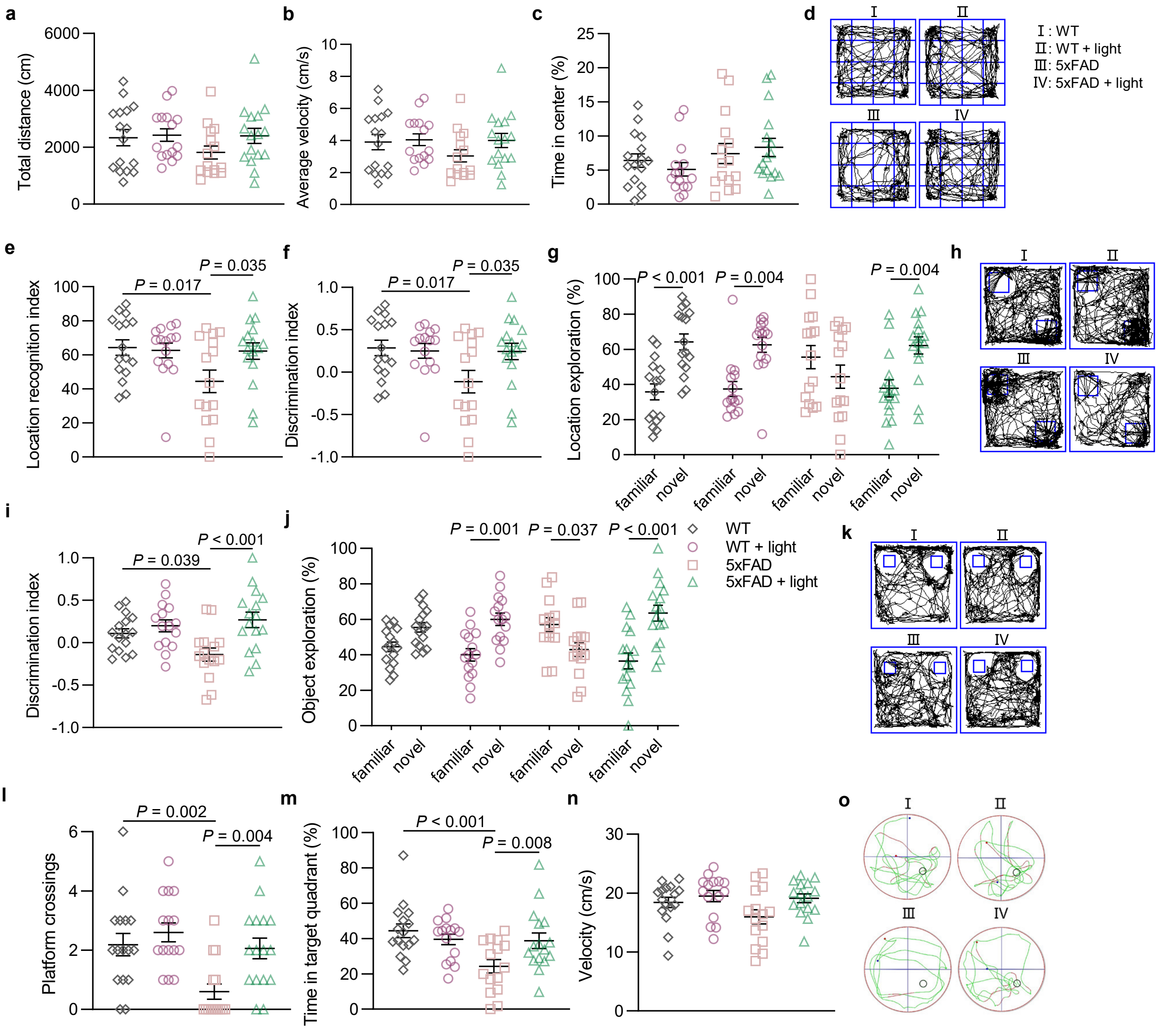
a
Total distance (cm)
6000
4000
2000
0
b
Average velocity (cm/s)
10
8
6
4
2
0
c
Time in center (%)
25
20
15
10
5
0
d
Ⅰ
Ⅱ
Ⅲ
Ⅳ
Ⅰ: WT
Ⅱ: WT + light
Ⅲ: 5xFAD
Ⅳ: 5xFAD + light
e
Location recognition index
P = 0.017
P = 0.035
100
80
60
40
20
0
f
Discrimination index
P = 0.017
P = 0.035
1.0
0.5
0.0
-0.5
-1.0
g
Location exploration (%)
P < 0.001
P = 0.004
P = 0.004
100
80
60
40
20
0
familiar
novel
familiar
novel
familiar
novel
familiar
novel
h
Ⅰ
Ⅱ
Ⅲ
Ⅳ
i
Discrimination index
P = 0.039
P < 0.001
1.0
0.5
0.0
-0.5
-1.0
j
Object exploration (%)
P = 0.001
P = 0.037
P < 0.001
100
80
60
40
20
0
familiar
novel
familiar
novel
familiar
novel
familiar
novel
WT
WT + light
5xFAD
5xFAD + light
k
Ⅰ
Ⅱ
Ⅲ
Ⅳ
l
Platform crossings
P = 0.002
P = 0.004
6
4
2
0
m
Time in target quadrant (%)
P < 0.001
P = 0.008
100
80
60
40
20
0
n
Velocity (cm/s)
30
20
10
0
o
Ⅰ
Ⅱ
Ⅲ
Ⅳ

Supplementary Fig. 2 | Effects of light on mobility and cognition in 5xFAD mice. a-d Total distance (**a**), average velocity (**b**), time spent in center (**c**) and representative test paths (**d**) of OF test. **e-h** Location recognition index (**e**), discrimination index (**f**), percentage of object exploration (**g**) and representative test paths (**h**) of NOL test. **i-k** Discrimination index (**i**), percentage of object exploration (**j**) and representative test paths (**k**) of NOR test. **l-o** Number of platform crossings (**l**), percentage of time spent in target quadrant (**m**), swimming velocity (**n**) and representative test paths (**o**) of MWM. n = 16 mice in WT and 5xFAD + light groups; n = 15 mice in WT + light and 5xFAD groups in data **a-c**, **e-g**, **i**, **j**, **l-n**. Data in **a-c**, **e-g**, **i**, **j**, **l-n** are presented as mean ± SEM, and analyzed by two-way ANOVA with Sidak's multiple comparison test for comparisons of multiple groups. All the measurements were taken from distinct samples. Source data are provided as a Source data file.

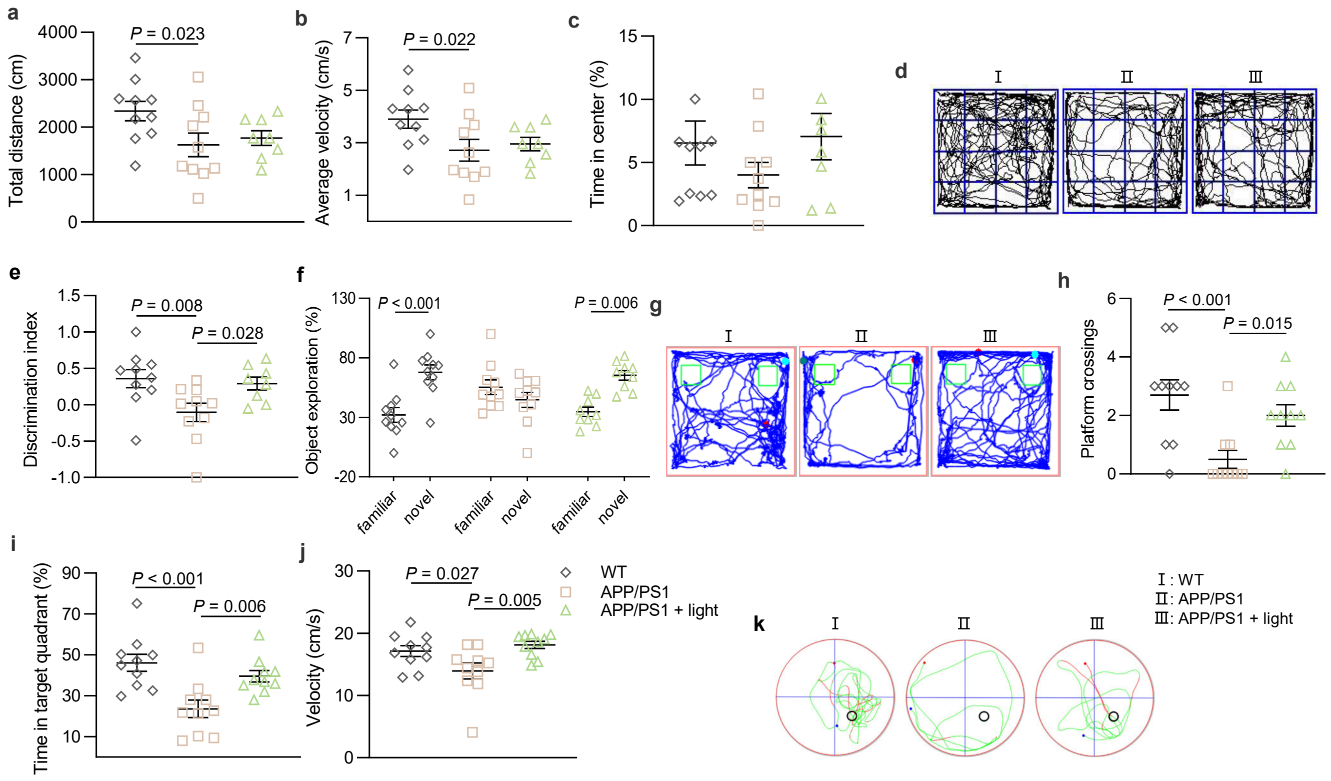
a
Total distance (cm)
P = 0.023
b
Average velocity (cm/s)
P = 0.022
c
Time in center (%)
d
Ⅰ
Ⅱ
Ⅲ
e
Discrimination index
P = 0.008
P = 0.028
f
Object exploration (%)
P < 0.001
P = 0.006
familiar
novel
familiar
novel
familiar
novel
g
Ⅰ
Ⅱ
Ⅲ
h
Platform crossings
P < 0.001
P = 0.015
i
Time in target quadrant (%)
P < 0.001
P = 0.006
j
Velocity (cm/s)
P = 0.027
P = 0.005
WT
APP/PS1
APP/PS1 + light
Ⅰ: WT
Ⅱ: APP/PS1
Ⅲ: APP/PS1 + light
k
Ⅰ
Ⅱ
Ⅲ

Supplementary Fig. 3 | Effects of light on mobility and cognition in APP/PS1 mice. a-d Total distance (**a**), average velocity (**b**), time spent in center (**c**) and representative test paths (**d**) of OF test. n = 10 mice in WT and APP/PS1 groups; n = 8 mice in APP/PS1 + light group. **e-g** Discrimination index (**e**), percentage of object exploration (**f**) and representative test paths (**g**) of NOR test. n = 10 mice in WT and APP/PS1 groups; n = 8 mice in APP/PS1 + light group. **h-k** Number of platform crossings (**h**), percentage of time spent in target quadrant (**i**), swimming velocity (**j**) and representative test paths (**k**) of MWM. n = 10 mice in each group. Data in **a-c**, **e**, **f**, **h-j** are presented as mean ± SEM, and analyzed by one-way ANOVA (**a-c**, **e**, **h-j**) or two-way ANOVA (**f**) with Sidak's multiple comparison test for comparisons of multiple groups. All the measurements were taken from distinct samples. Source data are provided as a Source data file.

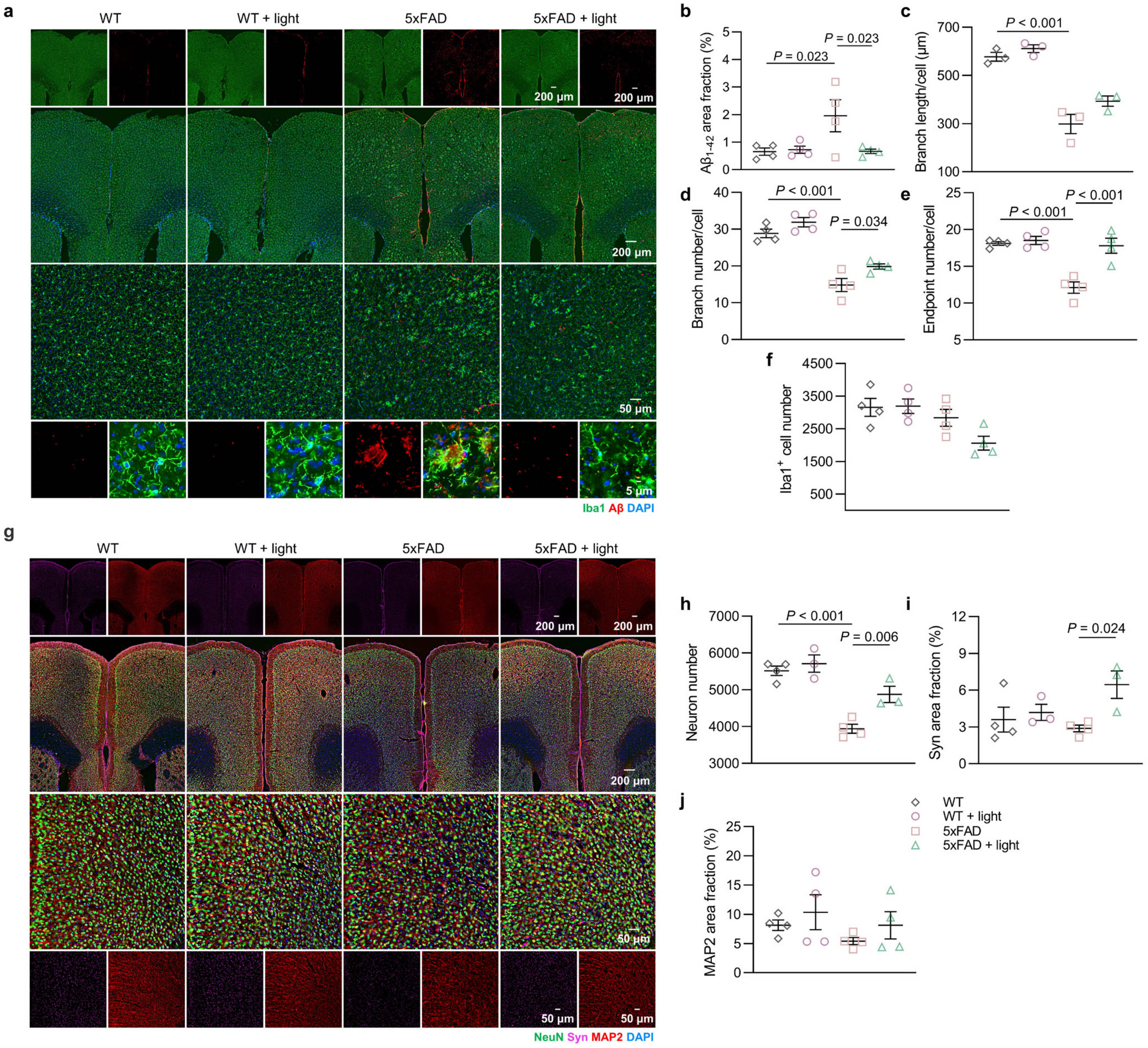

a
WT
WT + light
5xFAD
5xFAD + light
200 μm
200 μm
200 μm
50 μm
5 μm
Iba1 Aβ DAPI
b
Aβ1-42 area fraction (%)
P = 0.023
P = 0.023
c
Branch length/cell (μm)
P < 0.001
d
Branch number/cell
P < 0.001
P = 0.034
e
Endpoint number/cell
P < 0.001
P < 0.001
f
Iba1+ cell number
g
WT
WT + light
5xFAD
5xFAD + light
200 μm
200 μm
200 μm
50 μm
50 μm
50 μm
NeuN Syn MAP2 DAPI
h
Neuron number
P < 0.001
P = 0.006
i
Syn area fraction (%)
P = 0.024
j
MAP2 area fraction (%)
WT
WT + light
5xFAD
5xFAD + light

Supplementary Fig. 4 | Ameliorating effects of light on AD-associated pathology in prefrontal cortex (PFC) of 5xFAD mice. a Representative images of brain coronal sections in PFC stained with $A\beta_{1\text{-}42}$, Iba1 and DAPI. Scale bar = 200 µm, 50 µm or 5 µm. **b** Quantification of area fraction of $A\beta_{1\text{-}42}$ in PFC. **c-f** Quantification of branch length per cell (**c**), branch number per cell (**d**), endpoint number (**e**) and cell number (**f**) of $Iba1^{+}$ cells in PFC. n = 4 mice in each group in data **b**, **d-f**; n = 3 mice in each group in data **c**. **g** Representative images of brain coronal sections in PFC stained with NeuN, synaptophysin (Syn), microtubule associated protein 2 (MAP2) and DAPI. Scale bar = 200 µm or 50 µm. **h-j** Quantification of neuron number (**h**), area fraction of Syn (**i**) and MAP2 (**j**) in PFC. n = 4 mice in WT and 5xFAD groups, n = 3 mice in WT + light and 5xFAD + light groups in data **h**, **i**; n = 4 mice in each group in data **j**. Data in **b-f**, **h-j** are presented as mean ± SEM, and analyzed by two-way ANOVA with Sidak's multiple comparison test for comparisons of multiple groups. Source data are provided as a Source data file.

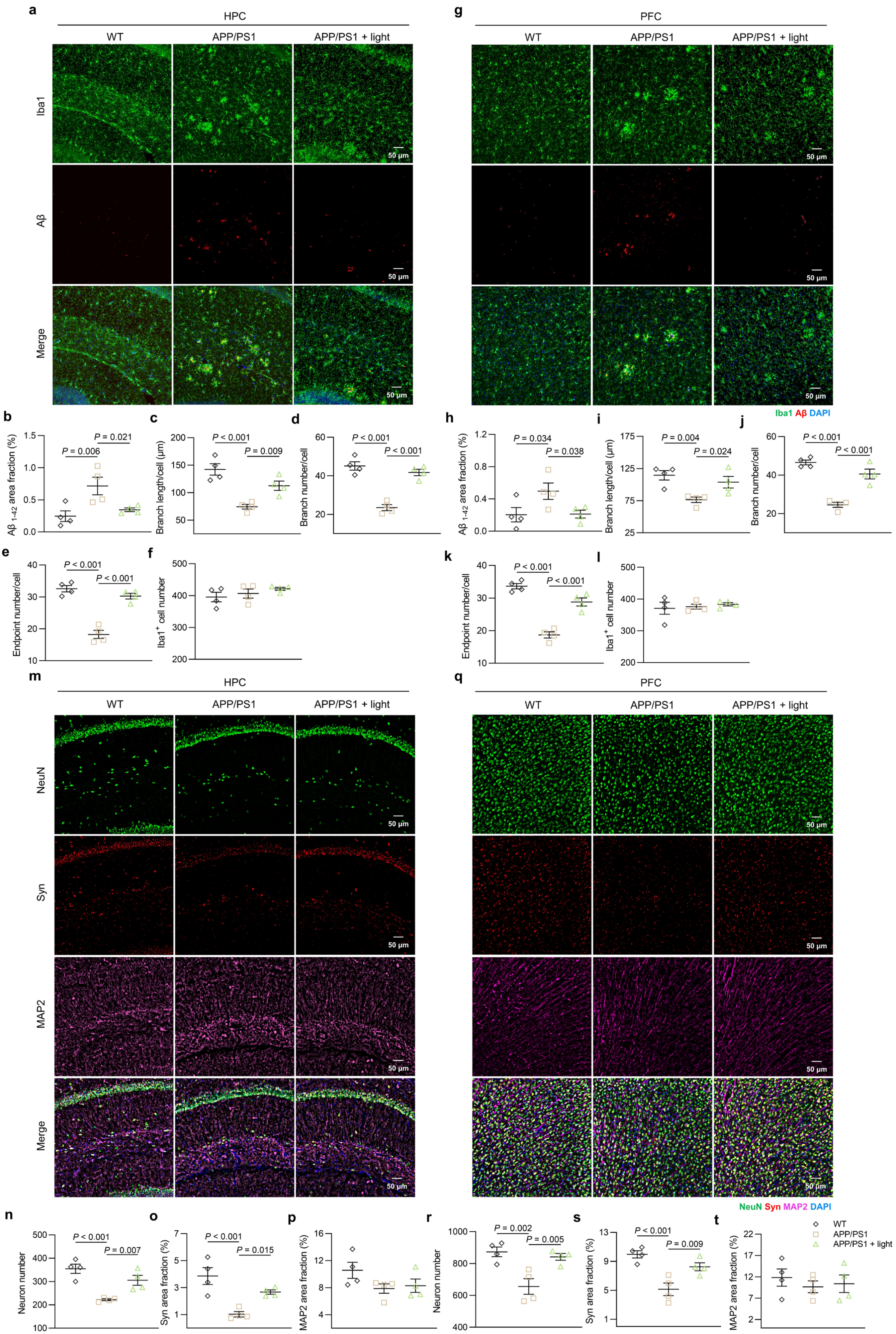

a
HPC
WT
APP/PS1
APP/PS1 + light
Iba1
Aβ
Merge
50 μm
g
PFC
Iba1 Aβ DAPI
b
Aβ 1-42 area fraction (%)
P = 0.006
P = 0.021
c
Branch length/cell (μm)
P < 0.001
P = 0.009
d
Branch number/cell
P < 0.001
P < 0.001
h
P = 0.034
P = 0.038
i
P = 0.004
P = 0.024
j
e
Endpoint number/cell
f
Iba1+ cell number
k
l
m
NeuN
Syn
MAP2
q
NeuN Syn MAP2 DAPI
n
Neuron number
P = 0.007
o
Syn area fraction (%)
P = 0.015
p
MAP2 area fraction (%)
r
P = 0.002
P = 0.005
s
P = 0.009
t
WT
APP/PS1
APP/PS1 + light

Supplementary Fig. 5 | Ameliorating effects of light on AD-associated pathology in HPC and PFC of APP/PS1 mice. a Representative images of brain coronal sections in HPC stained with $A\beta_{1-42}$, Iba1 and DAPI. Scale bar = 50 μm. **b** Quantification of area fraction of $A\beta_{1-42}$ in HPC. **c-f** Quantification of branch length per cell (**c**), branch number per cell (**d**), endpoint number (**e**) and cell number (**f**) of $Iba1^+$ cells in HPC. **g** Representative images of brain coronal sections in PFC stained with $A\beta_{1-42}$, Iba1 and DAPI. Scale bar = 50 μm. **h** Quantification of area fraction of $A\beta_{1-42}$ in PFC. **i-l** Quantification of branch length per cell (**i**), branch number per cell (**j**), endpoint number (**k**) and cell number (**l**) of $Iba1^+$ cells in PFC. **m**, Representative images of brain coronal sections in HPC stained with NeuN, Syn, MAP2 and DAPI. Scale bar = 50 μm. **n-p** Quantification of neuron number (**n**), area fraction of Syn (**o**) and MAP2 (**p**) in HPC. **q** Representative images of brain coronal sections in PFC stained with NeuN, Syn, MAP2 and DAPI. Scale bar = 50 μm. **r-t** Quantification of neuron number (**r**), area fraction of Syn (**s**) and MAP2 (**t**) in PFC. Data in **b-f**, **h-l**, **n-p**, **r-t** (n = 4 mice in each group) are presented as mean ± SEM, and analyzed by one-way ANOVA with Sidak's multiple comparison test for comparisons of multiple groups. Source data are provided as a Source data file.

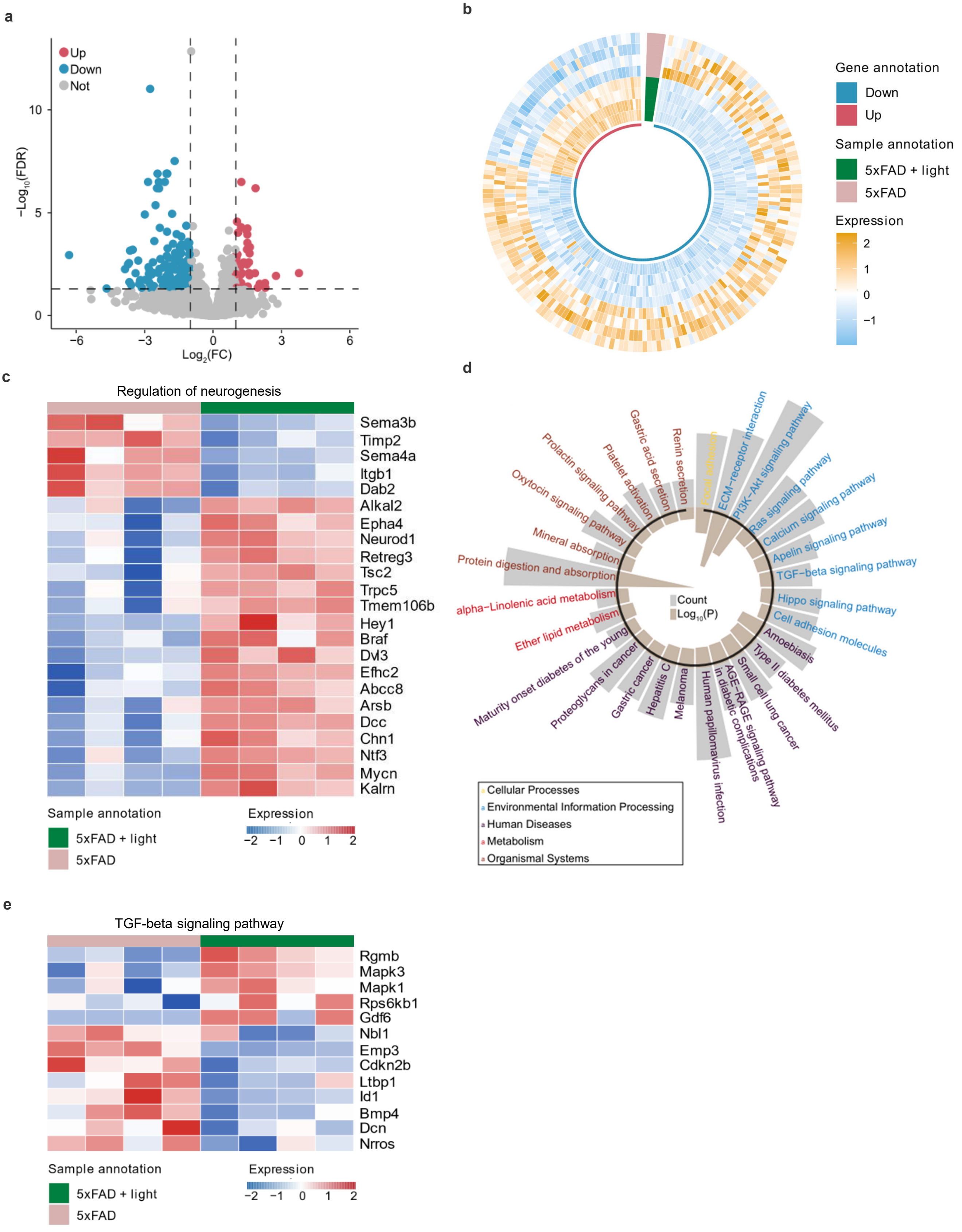
a
Up
Down
Not
−Log10(FDR)
Log2(FC)
b
Gene annotation
Down
Up
Sample annotation
5xFAD + light
5xFAD
Expression
c
Regulation of neurogenesis
Sema3b
Timp2
Sema4a
Itgb1
Dab2
Alkal2
Epha4
Neurod1
Retreg3
Tsc2
Trpc5
Tmem106b
Hey1
Braf
Dvl3
Efhc2
Abcc8
Arsb
Dcc
Chn1
Ntf3
Mycn
Kalrn
Sample annotation
5xFAD + light
5xFAD
Expression
d
Gastric acid secretion
Renin secretion
Focal adhesion
ECM−receptor interaction
PI3K−Akt signaling pathway
Ras signaling pathway
Calcium signaling pathway
Apelin signaling pathway
TGF−beta signaling pathway
Hippo signaling pathway
Cell adhesion molecules
Amoebiasis
Type II diabetes mellitus
Small cell lung cancer
AGE−RAGE signaling pathway in diabetic complications
Human papillomavirus infection
Melanoma
Hepatitis C
Gastric cancer
Proteoglycans in cancer
Maturity onset diabetes of the young
Ether lipid metabolism
alpha−Linolenic acid metabolism
Protein digestion and absorption
Mineral absorption
Oxytocin signaling pathway
Prolactin signaling pathway
Platelet activation
Count
Log10(P)
Cellular Processes
Environmental Information Processing
Human Diseases
Metabolism
Organismal Systems
e
TGF-beta signaling pathway
Rgmb
Mapk3
Mapk1
Rps6kb1
Gdf6
Nbl1
Emp3
Cdkn2b
Ltbp1
Id1
Bmp4
Dcn
Nrros
Sample annotation
5xFAD + light
5xFAD
Expression

Supplementary Fig. 6 | Improvement effects of light on gene expression of HPC in 5xFAD mice. a, **b** Volcano plot (**a**) and heatmap (**b**) showing up-regulated and down-regulated differentially expressed genes (DEGs) in HPC between light-treated 5xFAD group and 5xFAD group. Color scale bar values represent standardized log-transformed values across samples. **c** Heatmaps showing relative expression levels of DEGs involved in Regulation of neurogenesis. **d** Kyoto Encyclopedia of Genes and Genomes (KEGG) terms functional enrichment of DEGs of HPC tissues for group comparison, measured by the $\mathrm{Log}_{10}(P\ \mathrm{value})$. **e** Heatmaps showing relative expression levels of DEGs involved in TGF-beta signaling pathway. n = 4 mice in each group in data **a**-**e**. Source data are provided as a Source data file.

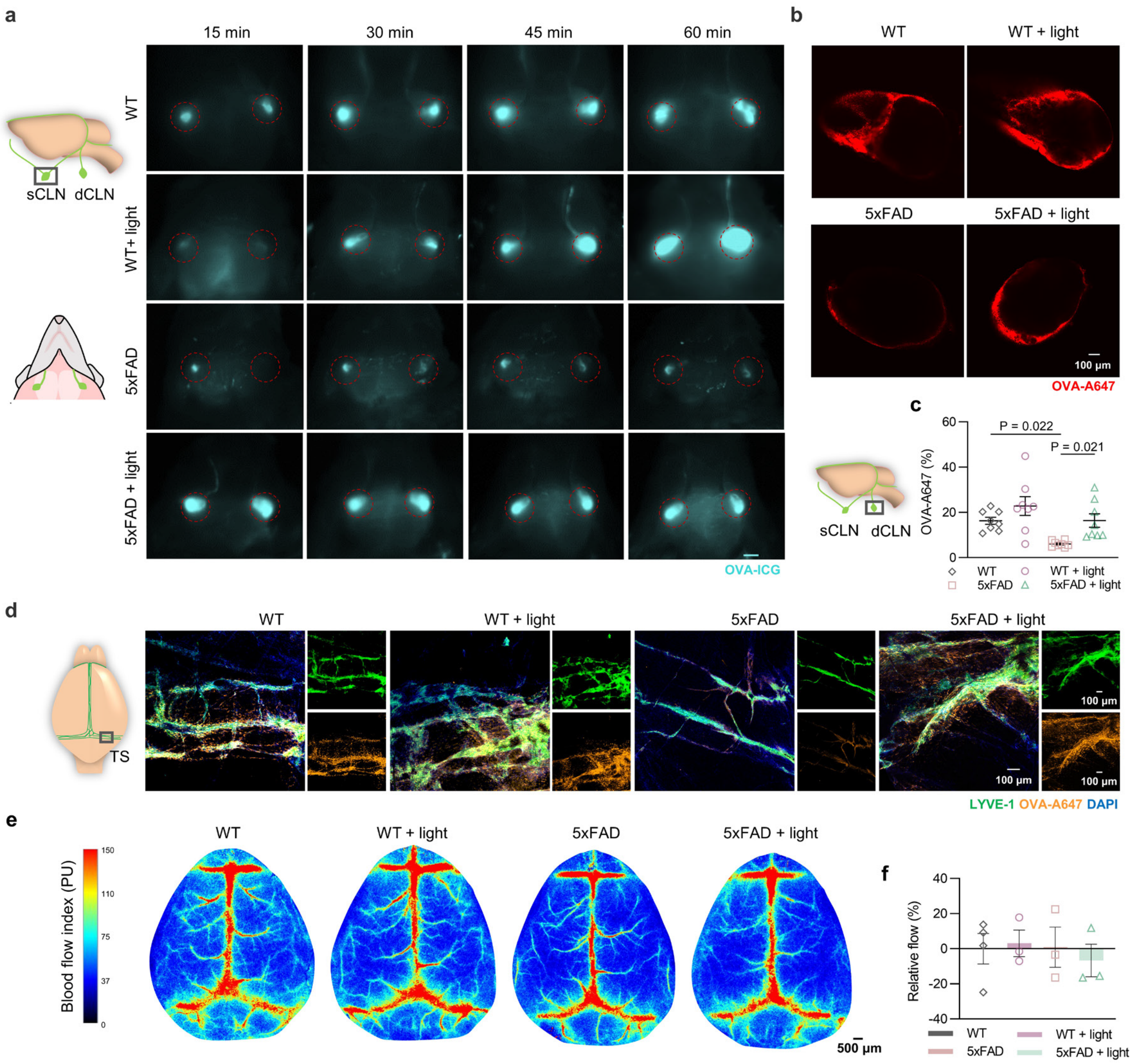
a
15 min
30 min
45 min
60 min
sCLN dCLN
WT
WT+ light
5xFAD
5xFAD + light
OVA-ICG
b
WT
WT + light
5xFAD
5xFAD + light
100 µm
OVA-A647
c
sCLN dCLN
OVA-A647 (%)
60
40
20
0
P = 0.022
P = 0.021
WT
WT + light
5xFAD
5xFAD + light
d
TS
WT
WT + light
5xFAD
5xFAD + light
100 µm
LYVE-1 OVA-A647 DAPI
e
Blood flow index (PU)
150
110
75
37
0
WT
WT + light
5xFAD
5xFAD + light
500 µm
f
Relative flow (%)
40
20
0
-20
-40
WT
WT + light
5xFAD
5xFAD + light

Supplementary Fig. 7 | Improvement effects of light on drainage function changes of mLVs in 5xFAD mice. a Representative images of OVA-ICG-accumulated superficial cervical lymph nodes (sCLNs) at different time after injection (i.c.m.) (from 3 replicates). Scale bar = 2 mm. **b** Representative images of OVA-A647-accumulated deep cervical lymph nodes (dCLNs) at 2 h after injection (i.c.m.) (from 2 replicates). Scale bar = 100 μm. **c** Quantification of fluorescence intensity of OVA-A647 in dCLNs. n = 8 mice in each group. **d** Representative images of OVA-A647-accumulated meninges in the transverse sinuse regions at 2 h after injection (i.c.m.), with LYVE-1 and DAPI staining (from 2 replicates). **e**, Representative images of blood flow obtained by LSCI of brain/meningeal vasculature of 5xFAD mice. **f**, Relative blood flow of 5xFAD mice after the treatments. Perfusion units, PU. n = 4 mice in WT group, n = 3 mice in WT + light, 5xFAD and 5xFAD + light groups. Data in **c**, **f** are presented as mean ± SEM, and analyzed by two-way ANOVA with Sidak's multiple comparison test for comparisons of multiple groups. Source data are provided as a Source data file.

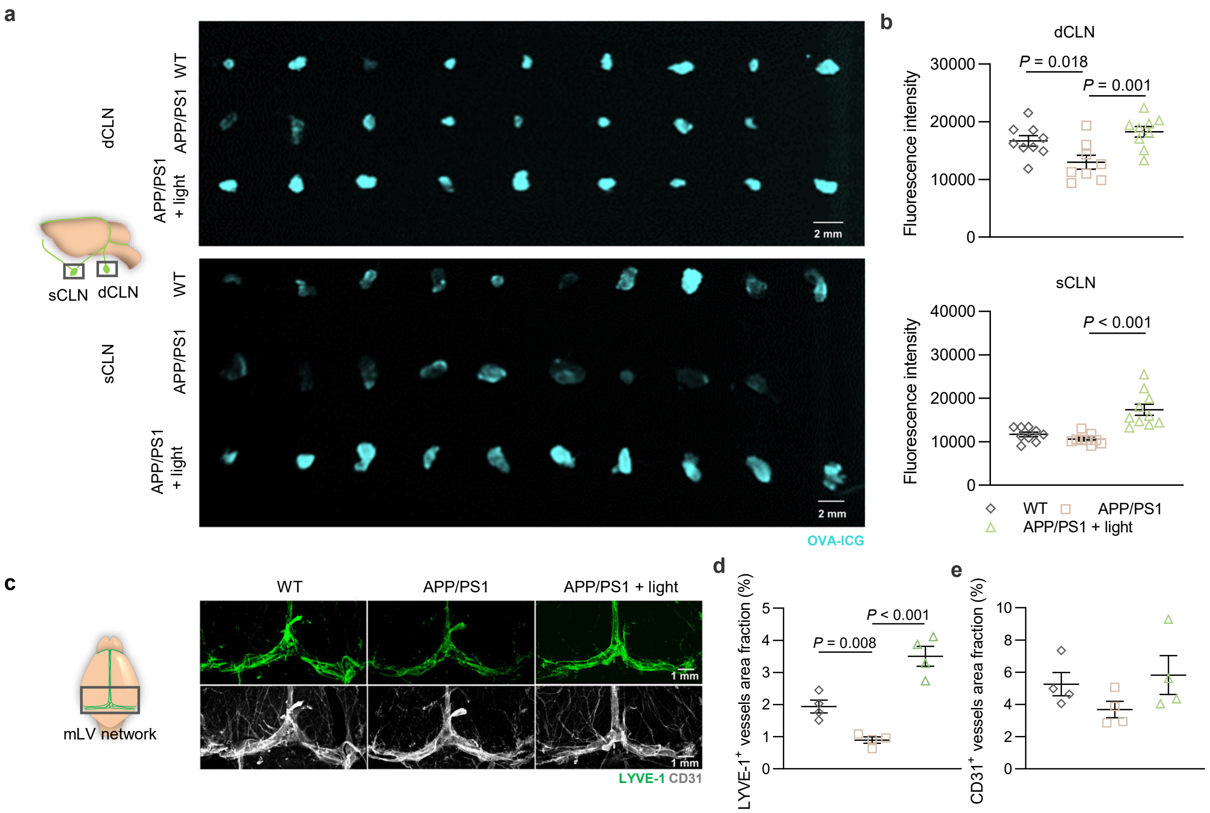

a
dCLN
WT
APP/PS1
APP/PS1 + light
2 mm
sCLN
sCLN dCLN
OVA-ICG
b
dCLN
P = 0.018
P = 0.001
Fluorescence intensity
30000
20000
10000
0
sCLN
P < 0.001
40000
WT
APP/PS1
APP/PS1 + light
c
mLV network
1 mm
LYVE-1 CD31
d
P = 0.008
LYVE-1+ vessels area fraction (%)
e
CD31+ vessels area fraction (%)

Supplementary Fig. 8 | Improvement effects of light on drainage function and structural changes of mLVs in APP/PS1 mice. a OVA-ICG-accumulated dCLNs and sCLNs at 2 h after injection (i.c.m.) (from 2 replicates). Scale bar = 2 mm. **b** Quantification of fluorescence intensity of OVA-ICG in dCLNs and sCLNs. n = 9 mice in WT and APP/PS1 + light groups, n = 8 mice in APP/PS1 group. **c** Representative images of meninges stained with LYVE-1 and CD31. Scale bar = 1 mm. **d**, **e** Quantification of area fraction of LYVE-1^{+} lymphatic vessels (**d**) and blood vessels (**e**) in meninges. n = 4 mice in each group. Data in **b**, **d**, **e** are presented as mean ± SEM, and analyzed by one-way ANOVA with Sidak's multiple comparison test for comparisons of multiple groups. Source data are provided as a Source data file.

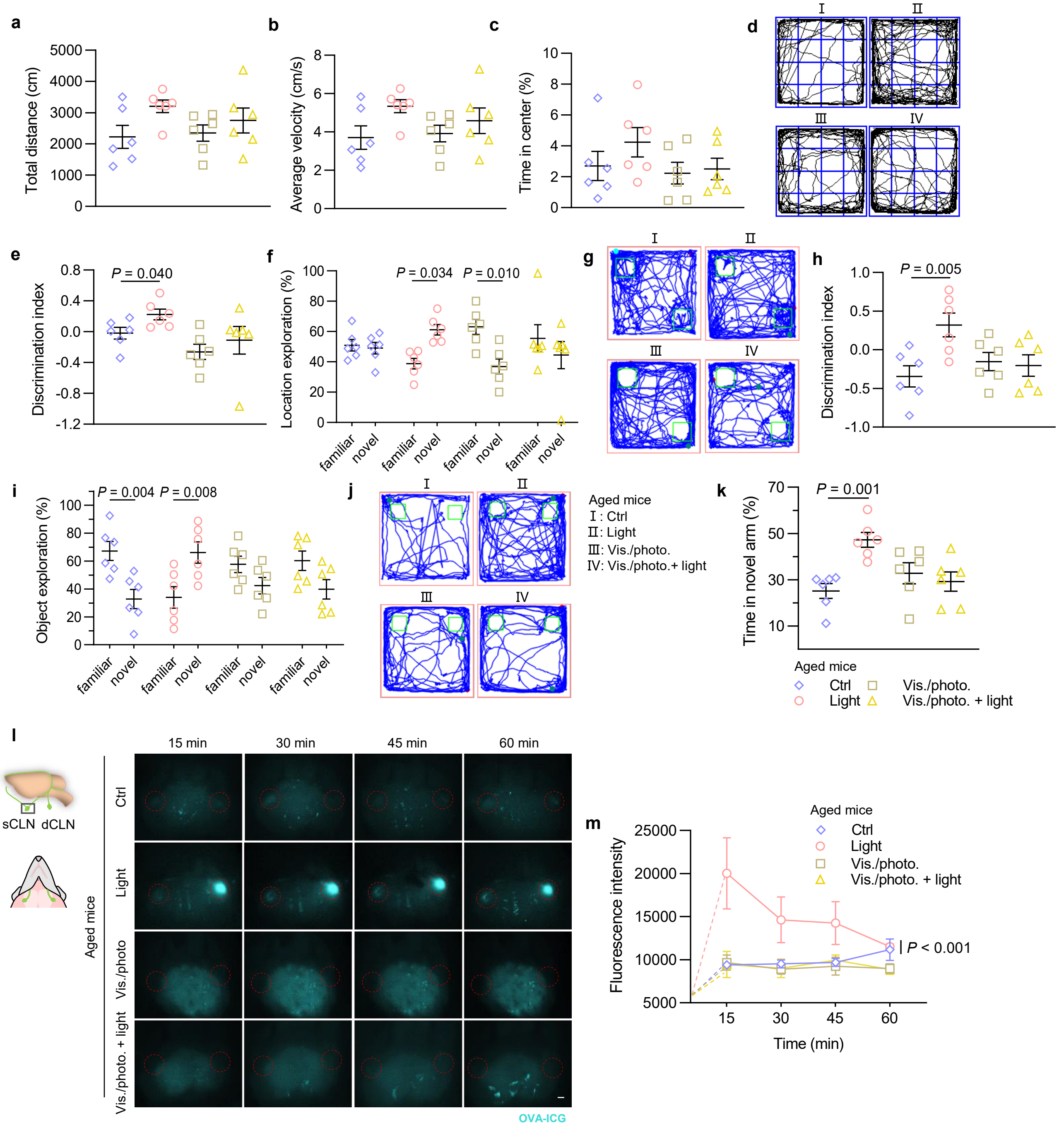
a
Total distance (cm)
b
Average velocity (cm/s)
c
Time in center (%)
d
e
Discrimination index
P = 0.040
f
Location exploration (%)
P = 0.034
P = 0.010
familiar
novel
g
h
Discrimination index
P = 0.005
i
Object exploration (%)
P = 0.004
P = 0.008
j
Aged mice
Ⅰ: Ctrl
Ⅱ: Light
Ⅲ: Vis./photo.
Ⅳ: Vis./photo.+ light
k
Time in novel arm (%)
P = 0.001
Aged mice
Ctrl
Light
Vis./photo.
Vis./photo. + light
l
sCLN
dCLN
15 min
30 min
45 min
60 min
Ctrl
Light
Vis./photo
Vis./photo. + light
Aged mice
OVA-ICG
m
Fluorescence intensity
Time (min)
P < 0.001

Supplementary Fig. 9 | Effects of light on cognition and lymphatic drainage in mLV-ablated aged mice. a-d Total distance (**a**), average velocity (**b**), time spent in center (**c**) and representative test paths (**d**) of OF test. **e-g** Discrimination index (**e**), percentage of object exploration (**f**) and representative test paths (**g**) of NOL test. **h-j** Discrimination index (**h**), percentage of object exploration (**i**) and representative test paths (**j**) of NOR test. **k** Percentage of time spent in novel arm of Y-maze test. n = 6 mice in each group in data **a-c**, **e**, **f**, **h**, **i**, **k**. **l** Representative images of sCLNs with OVA-ICG accumulation (marked with red circles) at different time points after injection (i.c.m.). Scale bar = 1 mm. **m** Quantification of fluorescence intensity of OVA-ICG in sCLNs. n = 4 mice in each group. Data in **a-c**, **e**, **f**, **h**, **i**, **k**, **m** are presented as mean ± SEM, and analyzed by two-way ANOVA with Sidak's multiple comparison test for comparisons of multiple groups. Source data are provided as a Source data file.

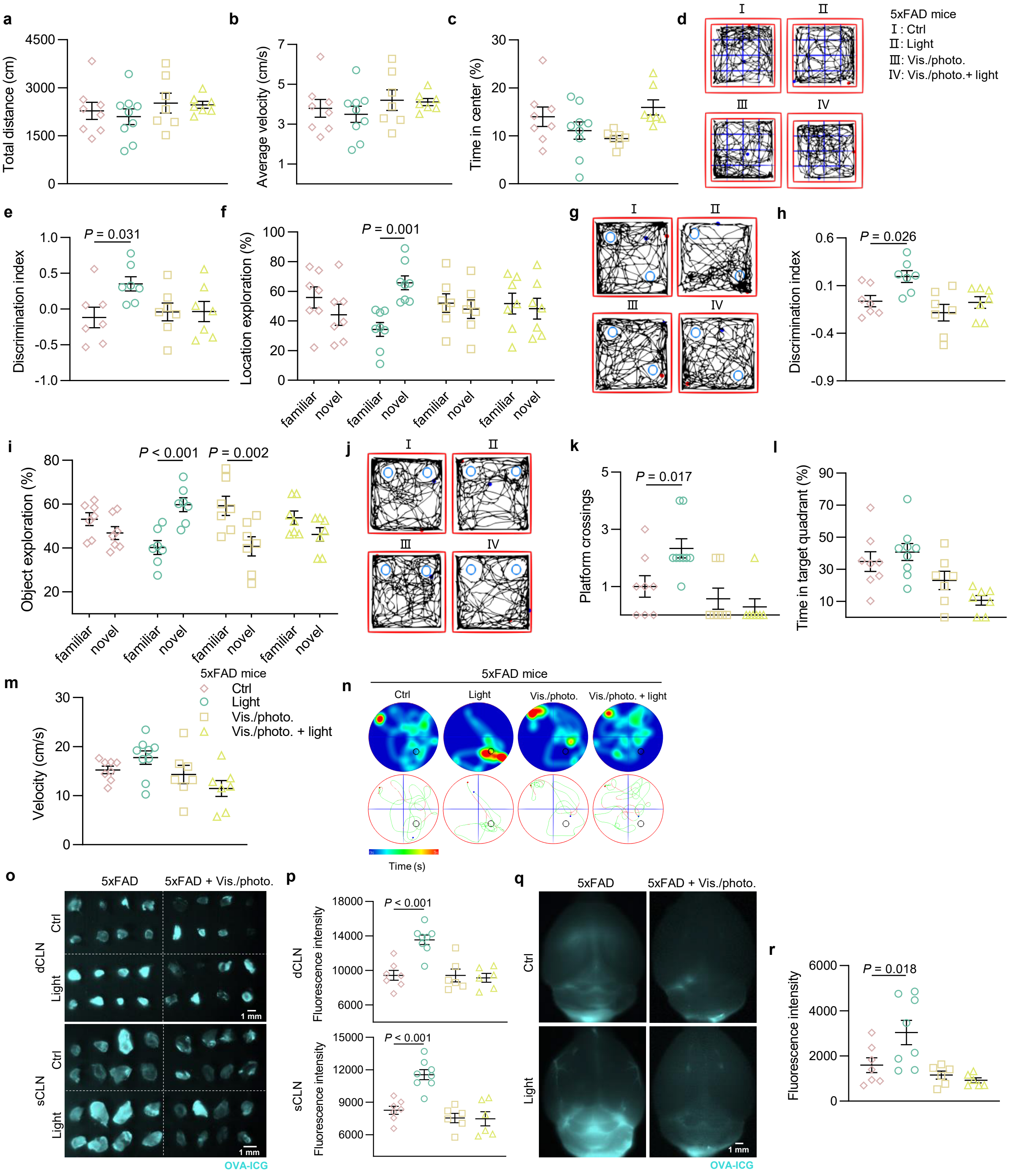

a
Total distance (cm)
b
Average velocity (cm/s)
c
Time in center (%)
d
5xFAD mice
Ⅰ: Ctrl
Ⅱ: Light
Ⅲ: Vis./photo.
Ⅳ: Vis./photo.+ light
e
Discrimination index
P = 0.031
f
Location exploration (%)
P = 0.001
familiar
novel
g
h
Discrimination index
P = 0.026
i
Object exploration (%)
P < 0.001
P = 0.002
j
k
Platform crossings
P = 0.017
l
Time in target quadrant (%)
m
Velocity (cm/s)
5xFAD mice
Ctrl
Light
Vis./photo.
Vis./photo. + light
n
5xFAD mice
Ctrl
Light
Vis./photo.
Vis./photo. + light
Time (s)
o
5xFAD
5xFAD + Vis./photo.
dCLN
sCLN
Ctrl
Light
1 mm
OVA-ICG
p
dCLN
sCLN
Fluorescence intensity
P < 0.001
q
5xFAD
5xFAD + Vis./photo.
Ctrl
Light
1 mm
OVA-ICG
r
Fluorescence intensity
P = 0.018

Supplementary Fig. 10 | Effects of light on cognition and lymphatic drainage function in mLV-ablated 5xFAD mice. a-d Total distance (**a**), average velocity (**b**), time spent in center (**c**) and representative test paths (**d**) of OF test. n = 8 mice in Ctrl group, n = 9 mice in light group, n = 7 mice in Vis./photo. and Vis./photo. + light groups. **e-g** Discrimination index (**e**), percentage of object exploration (**f**) and representative test paths (**g**) of NOL test. n = 7 mice in each group. **h-j** Discrimination index (**h**), percentage of object exploration (**i**) and representative test paths (**j**) of NOR test. n = 7 mice in each group. **k-n** Number of platform crossings (**k**), percentage of time spent in target quadrant (**l**), swimming velocity (**m**) and representative test paths (**n**) of MWM. n = 8 mice in Ctrl group, n = 9 mice in Light group, n = 7 mice in Vis./photo. and Vis./photo. + light groups. **o** OVA-ICG-accumulated dCLNs and sCLNs at 2 h after injection (i.c.m.) brain (from 2 replicates). Scale bar = 1 mm. **p** Quantification of fluorescence intensity of OVA-ICG in dCLNs and sCLNs. **q** Representative images of the brains with OVA-ICG influx after injection (i.c.m.) (from 2 replicates). Scale bar =1 mm. **r** Quantification of fluorescence intensity of OVA-ICG influx in the brain. n = 7 mice in Ctrl group, n = 8 mice in Light group, n = 6 mice in Vis./photo. and Vis./photo. + light groups in data **o-r**. Data in **a-c**, **e**, **f**, **h**, **i**, **k-m**, **p**, **r** are presented as mean ± SEM, and analyzed by two-way ANOVA with Sidak's multiple comparison test for comparisons of multiple groups. Source data are provided as a Source data file.

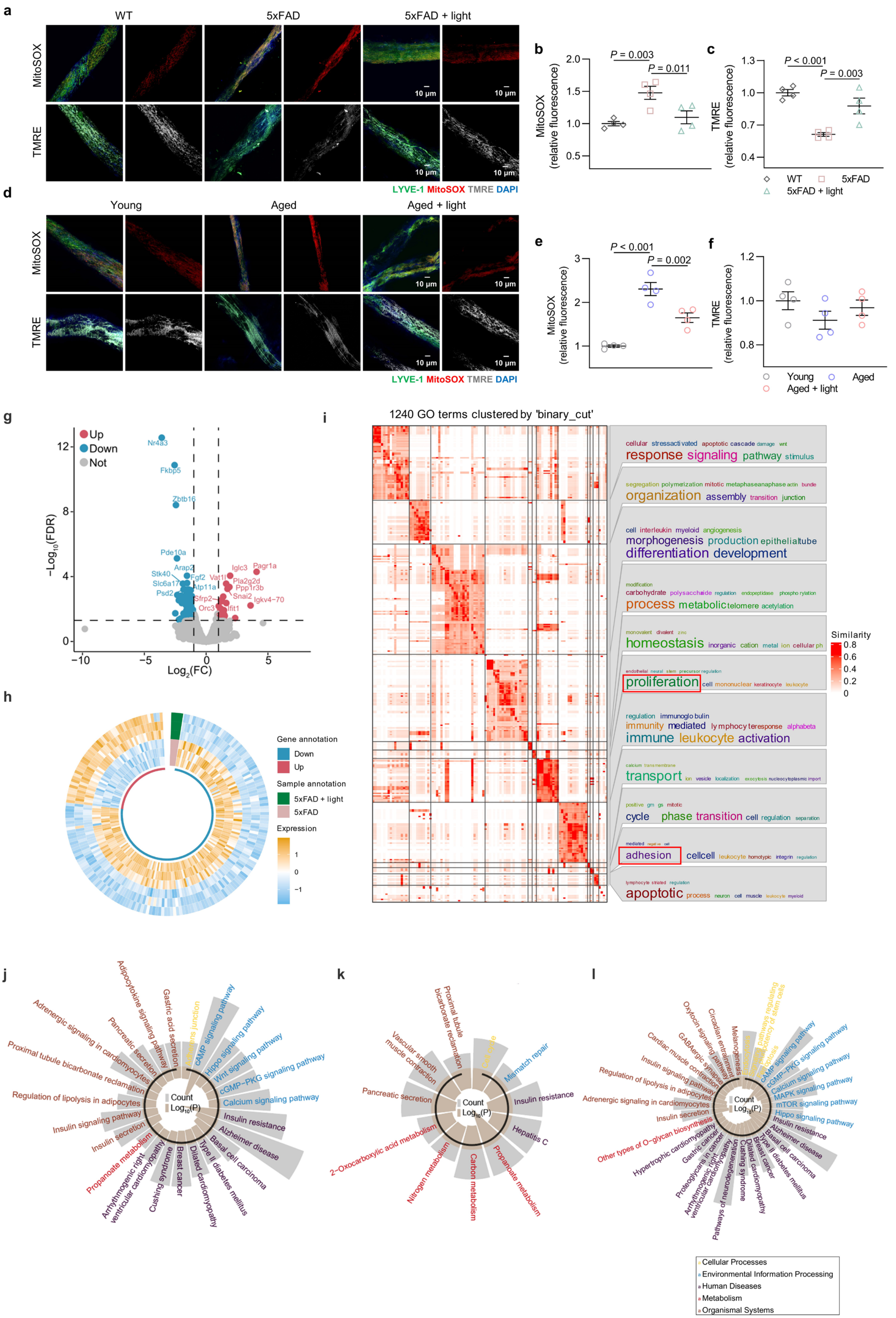

a
WT
5xFAD
5xFAD + light
MitoSOX
TMRE
10 µm
LYVE-1 MitoSOX TMRE DAPI
b
MitoSOX (relative fluorescence)
P = 0.003
P = 0.011
c
TMRE (relative fluorescence)
P < 0.001
P = 0.003
WT
5xFAD
5xFAD + light
d
Young
Aged
Aged + light
e
P < 0.001
P = 0.002
f
Young
Aged
Aged + light
g
Up
Down
Not
−Log10(FDR)
Log2(FC)
Nr4a3
Fkbp5
Zbtb16
Pde10a
Arap2
Stk40
Slc6a17
Psd2
Fgf2
Atp11a
Vat1l
Iglc3
Pagr1a
Pla2g2d
Ppp1r3b
Snai2
Igkv4-70
Sfrp2
Orc3
Ifit1
h
Gene annotation
Down
Up
Sample annotation
5xFAD + light
5xFAD
Expression
i
1240 GO terms clustered by 'binary_cut'
Similarity
response signaling pathway stimulus
organization assembly transition junction
morphogenesis production epithelial tube
differentiation development
process metabolic telomere acetylation
homeostasis inorganic cation metal ion cellular ph
proliferation
immunity mediated lymphocyte response alphabeta
immune leukocyte activation
transport
cycle phase transition cell regulation separation
adhesion cellcell leukocyte homotypic integrin regulation
apoptotic process neuron cell muscle leukocyte myeloid
j
Count
Log10(P)
Adipocytokine signaling pathway
Gastric acid secretion
Adherens junction
cAMP signaling pathway
Hippo signaling pathway
Wnt signaling pathway
cGMP-PKG signaling pathway
Calcium signaling pathway
Insulin resistance
Alzheimer disease
Basal cell carcinoma
Type II diabetes mellitus
Dilated cardiomyopathy
Breast cancer
Cushing syndrome
Arrhythmogenic right ventricular cardiomyopathy
Propanoate metabolism
Insulin secretion
Insulin signaling pathway
Regulation of lipolysis in adipocytes
Proximal tubule bicarbonate reclamation
Adrenergic signaling in cardiomyocytes
Pancreatic secretion
k
Proximal tubule bicarbonate reclamation
Cell cycle
Mismatch repair
Insulin resistance
Hepatitis C
Propanoate metabolism
Carbon metabolism
Nitrogen metabolism
2-Oxocarboxylic acid metabolism
Pancreatic secretion
Vascular smooth muscle contraction
l
Oxytocin signaling pathway
Circadian entrainment
Melanogenesis
Cardiac muscle contraction
GABAergic synapse
cAMP signaling pathway
cGMP-PKG signaling pathway
Calcium signaling pathway
MAPK signaling pathway
mTOR signaling pathway
Hippo signaling pathway
Insulin resistance
Alzheimer disease
Basal cell carcinoma
Type II diabetes mellitus
Breast cancer
Dilated cardiomyopathy
Cushing syndrome
Pathways of neurodegeneration
Arrhythmogenic right ventricular cardiomyopathy
Proteoglycans in cancer
Gastric cancer
Hypertrophic cardiomyopathy
Other types of O-glycan biosynthesis
Insulin secretion
Adrenergic signaling in cardiomyocytes
Regulation of lipolysis in adipocytes
Insulin signaling pathway
Cellular Processes
Environmental Information Processing
Human Diseases
Metabolism
Organismal Systems

Supplementary Fig. 11 | Improvement effects of light on gene expression of meninges in 5xFAD mice. a Representative images of mLVs of 5xFAD mice stained with LYVE-1, mitochondrial superoxide indicator (MitoSOX) and tetramethylrhodamine (TMRE) brain (from 2 replicates). Scale bar = 10 μm. **b** Quantification of mitochondrial superoxide in mLECs measured by MitoSOX fluorescent. **c** Quantification of mitochondrial membrane potential in mLECs measured by TMRE fluorescent brain. **d** Representative images of mLVs of aged mice stained with LYVE-1, MitoSOX and TMRE (from 2 replicates). Scale bar = 10 μm. **e** Quantification of mitochondrial superoxide in mLECs measured by MitoSOX fluorescent. **f** Quantification of mitochondrial membrane potential in mLECs measured by TMRE fluorescent. n = 4 mice in each group in data **b**, **c**, **e**, **f**. **g-h** Volcano plot (**g**) and heatmap (**h**) showing up-regulated and down-regulated DEGs in meninges of 5xFAD mice received light treatment compared with sham group. Color scale bar values represent standardized log-transformed values across samples. **i** Similarity matrix illustrating clustering of up-regulated GO biological process terms, with the right word clouds containing overrepresented keywords from the significant GO terms. **j-l** KEGG functional enrichment of all DEGs (**j**), up-regulated DEGs (**k**) and down-regulated DEGs (**l**) in meninges for group comparison of light-treated 5xFAD group versus 5xFAD group, measured by the $\mathrm{Log}_{10}(P \text{ value})$. n = 3 mice in each group in data **g-l**. Data in **b**, **c**, **e**, **f** are presented as mean ± SEM, and analyzed by one-way ANOVA with Sidak's multiple comparison test for comparisons of multiple groups. Source data are provided as a Source data file.

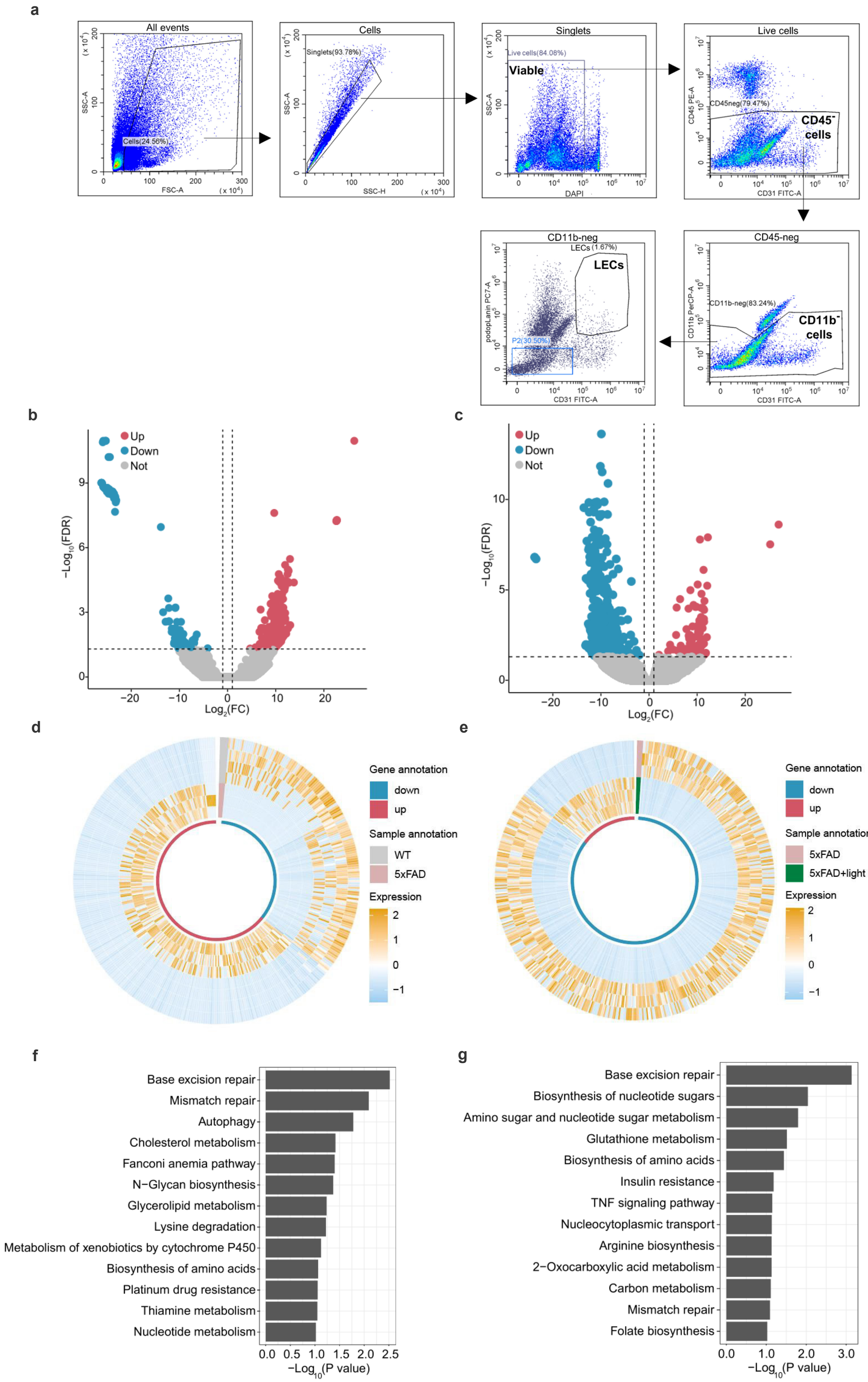
a
All events
Cells(24.56%)
SSC-A
FSC-A
Cells
Singlets(93.78%)
SSC-H
Singlets
Live cells(84.08%)
Viable
DAPI
Live cells
CD45neg(79.47%)
CD45⁻ cells
CD45 PE-A
CD31 FITC-A
CD45-neg
CD11b-neg(83.24%)
CD11b⁻ cells
CD11b PerCP-A
CD11b-neg
LECs (1.67%)
LECs
P2(30.50%)
podopLanin PC7-A
b
Up
Down
Not
−Log10(FDR)
Log2(FC)
c
Up
Down
Not
−Log10(FDR)
Log2(FC)
d
Gene annotation
down
up
Sample annotation
WT
5xFAD
Expression
e
Gene annotation
down
up
Sample annotation
5xFAD
5xFAD+light
Expression
f
Base excision repair
Mismatch repair
Autophagy
Cholesterol metabolism
Fanconi anemia pathway
N−Glycan biosynthesis
Glycerolipid metabolism
Lysine degradation
Metabolism of xenobiotics by cytochrome P450
Biosynthesis of amino acids
Platinum drug resistance
Thiamine metabolism
Nucleotide metabolism
−Log10(P value)
g
Base excision repair
Biosynthesis of nucleotide sugars
Amino sugar and nucleotide sugar metabolism
Glutathione metabolism
Biosynthesis of amino acids
Insulin resistance
TNF signaling pathway
Nucleocytoplasmic transport
Arginine biosynthesis
2−Oxocarboxylic acid metabolism
Carbon metabolism
Mismatch repair
Folate biosynthesis
−Log10(P value)

Supplementary Fig. 12 | Improvement effects of light on gene expression of mLECs in 5xFAD mice. a Representative plots showing the gating strategy for mLEC isolation from the meninges of mice in different groups by fluorescence-activated cell sorting (FACS). **b**, **c** Volcano plots of DEGs of mLECs between 5xFAD group versus WT group (**b**) and light-treated 5xFAD group versus 5xFAD group (**c**). **d**, **e** Heatmaps of DEGs of mLECs between 5xFAD group versus WT group (**d**) and light-treated 5xFAD group versus 5xFAD group (**e**). Color scale bar values represent standardized log-transformed values across samples. **f**, **g** KEGG functional enrichment of down-regulated genes between 5xFAD group versus WT group (**f**) and up-regulated genes between light-treated 5xFAD group versus 5xFAD group (**g**), measured by the $Log_{10}(P$ value). n = 4 mice in WT group, n = 3 mice in 5xFAD and 5xFAD + light groups in data **a**-**g**. Source data are provided as a Source data file.

Supplementary Table 1 Expression of genes involved in tight junction and cell adhesion in meninges between light-treated AD group versus AD group

**Gene ID**	***P* value**	**Log_2(FC)**	**GO**
Ocln	0.00013	1.43058	Bicellular tight junction assembly
Pcdhgb5	0.03884	1.63260	Cell adhesion
Pcdhgb6	0.01339	1.10344	Cell adhesion
Pcdhgb7	0.02802	1.12507	Cell adhesion
Pcdhga8	0.01055	1.53158	Cell adhesion
Hapln2	0.02410	4.43891	Cell adhesion

The statistical significance in Supplementary Table 1 was assessed with DESeg2 using the Wald test, without any adjustments for multiple comparisons. n=3 mice in each group. The statistical tests involved one-sided analysis. Source data are provided as a Source Data file.
