# Supplementary material for: Non-invasive modulation of meningeal lymphatics ameliorates ageing and Alzheimer’s disease-associated pathology and cognition in mice
Source: Nat Commun. 2024 Feb 16;15:1453. doi: 10.1038/s41467-024-45656-7 (PMC10873306; doi:10.1038/s41467-024-45656-7)
Supplement: Supplementary file 2 — Reporting Summary [file 41467_2024_45656_MOESM2_ESM.pdf]

nature portfolio

Corresponding author(s): Feifan Zhou
Last updated by author(s): Jan 19, 2024

Reporting Summary

Nature Portfolio wishes to improve the reproducibility of the work that we publish. This form provides structure for consistency and transparency in reporting. For further information on Nature Portfolio policies, see our Editorial Policies and the Editorial Policy Checklist.

Statistics

For all statistical analyses, confirm that the following items are present in the figure legend, table legend, main text, or Methods section.

n/a	Confirmed	
☐	☒	The exact sample size (*n*) for each experimental group/condition, given as a discrete number and unit of measurement
☐	☒	A statement on whether measurements were taken from distinct samples or whether the same sample was measured repeatedly
☐	☒	The statistical test(s) used AND whether they are one- or two-sided<br>*Only common tests should be described solely by name; describe more complex techniques in the Methods section.*
☐	☒	A description of all covariates tested
☐	☒	A description of any assumptions or corrections, such as tests of normality and adjustment for multiple comparisons
☐	☒	A full description of the statistical parameters including central tendency (e.g. means) or other basic estimates (e.g. regression coefficient) AND variation (e.g. standard deviation) or associated estimates of uncertainty (e.g. confidence intervals)
☐	☒	For null hypothesis testing, the test statistic (e.g. *F*, *t*, *r*) with confidence intervals, effect sizes, degrees of freedom and *P* value noted<br>*Give P values as exact values whenever suitable.*
☒	☐	For Bayesian analysis, information on the choice of priors and Markov chain Monte Carlo settings
☒	☐	For hierarchical and complex designs, identification of the appropriate level for tests and full reporting of outcomes
☒	☐	Estimates of effect sizes (e.g. Cohen's *d*, Pearson's *r*), indicating how they were calculated

Our web collection on statistics for biologists contains articles on many of the points above.

Software and code

Policy information about availability of computer code

Data collection	Immunofluorescent imaging: Olympus FLUOVIEW FV3000 Microscope (Olympus, Japan) and Olympus SLIDEVIEW VS200 Slide Scaner (Olympus, Japan).<br>NIR-II fluorescence imaging: MARS in vivo imaging system (Artemis Intelligent Imaging, China).<br>Blood flow imaging: laser speckle contrast imaging microscope (Simopto, China).<br>TEM imaging: transmission electron microscope (Tecnai G2 Spirit, FEI, USA).<br>Temperature monitoring: NIR thermal camera (226S, FOTRIC, China).<br>Cell sorting: flow cytometry (MoFlo Astrios, Beckman Coulter, USA).<br>Behavioral tests: Visutrack software (version 3.0, Xinruan, China) and DigBehv (version 4.1, Jiliang, China).
Data analysis	Statistical analysis and graph generation: Graphpad Prism (version 8.4.2).<br>Image export and immunofluorescent analysis: OLYMPUS OlyVIA (version 4.1) and Fiji software (version 1.53q).<br>Fluorescence intensity analysis: Fiji software (version 1.53q) and LightField (version 6.14).<br>Blood flow analysis: SIM BFI software (version 3.1.45).<br>Temperature analysis: AnalyzIR (version 4.3.2.71).<br>FACS analysis: CytExpert (version 2.5).<br>RNA-seq data analyses: All RNA-seq data analyses were performed in the statistical software R (version 4.3.1). Differential expression analysis was conducted and visualized using the R package DESeq2 (version 1.40.2) and ggplot2 (version 3.4.2). Those genes with a fold change value greater than 2.0 or lower than 0.5 and false discovery rate (FDR) -corrected $P < 0.05$ were considered significantly differentially expressed genes (DEGs). The Kyoto Encyclopedia of Genes and Genomes (KEGG) pathway enrichment analysis and Gene Ontology (GO) term enrichment analysis were carried out with the clusterProfiler software (version 4.8.3). GO terms similarity based on similarity matrices of functional terms

was performed using R packages simplifyEnrichment (version 1.10.0). Gene Set Enrichment Analysis (GSEA) was performed by ranking all genes based on their level of difference using R package clusterProfiler (version 4.8.3) and visualized using R package enrichplot (version 1.20.3). The hallmark gene sets served as reference sets for GSEA were obtained from the Molecular Signatures Database (https://www.gsea-msigdb.org/gsea/msigdb). Heatmap were visualized using ggplot2 (version 3.4.2) and pheatmap (version 1.0.12).

For manuscripts utilizing custom algorithms or software that are central to the research but not yet described in published literature, software must be made available to editors and reviewers. We strongly encourage code deposition in a community repository (e.g. GitHub). See the Nature Portfolio guidelines for submitting code & software for further information.

Data

Policy information about availability of data

All manuscripts must include a data availability statement. This statement should provide the following information, where applicable:

- Accession codes, unique identifiers, or web links for publicly available datasets
- A description of any restrictions on data availability
- For clinical datasets or third party data, please ensure that the statement adheres to our policy

The data that support the findings of this study are available within the article, the Supplementary Information files and the Source Data files. Source data are provided with this paper. The RNA sequencing data generated for this study can be found in the GEO repository under accession number GSE245658 (https://www.ncbi.nlm.nih.gov/geo/query/acc.cgi?acc=GSE245658). The hallmark gene sets served as reference sets for GSEA in RNA-seq analysis were obtained from the Molecular Signatures Database (https://www.gsea-msigdb.org/gsea/msigdb/).

Research involving human participants, their data, or biological material

Policy information about studies with human participants or human data. See also policy information about sex, gender (identity/presentation), and sexual orientation and race, ethnicity and racism.

Reporting on sex and gender	No human research participants were included in this study.
Reporting on race, ethnicity, or other socially relevant groupings	No human research participants were included in this study.
Population characteristics	No human research participants were included in this study.
Recruitment	No human research participants were included in this study.
Ethics oversight	No human research participants were included in this study.

Note that full information on the approval of the study protocol must also be provided in the manuscript.

Field-specific reporting

Please select the one below that is the best fit for your research. If you are not sure, read the appropriate sections before making your selection.

[x] Life sciences [ ] Behavioural & social sciences [ ] Ecological, evolutionary & environmental sciences

For a reference copy of the document with all sections, see nature.com/documents/nr-reporting-summary-flat.pdf

Life sciences study design

All studies must disclose on these points even when the disclosure is negative.

Sample size	The sample size in this study were estimated based on the similar experiments published in Nature communications 2020 (DOI: 10.1038/s41467-020-16851-z) and Nature 2019 (DOI: 10.1038/s41586-019-1419-5).
Data exclusions	No data were excluded.
Replication	All the experiments were successfully performed at least twice times to ensure reproducibility of the results.
Randomization	Mice were randomized upon arrival and were randomly assigned to different groups immediately before the start of the experiments.
Blinding	Experimenters were blind to the group allocation of mice in all the experiments during data collection and analysis.

Reporting for specific materials, systems and methods

We require information from authors about some types of materials, experimental systems and methods used in many studies. Here, indicate whether each material, system or method listed is relevant to your study. If you are not sure if a list item applies to your research, read the appropriate section before selecting a response.

Materials & experimental systems

n/a	Involved in the study	
	x	Antibodies
x		Eukaryotic cell lines
x		Palaeontology and archaeology
	x	Animals and other organisms
x		Clinical data
x		Dual use research of concern
x		Plants

Methods

n/a	Involved in the study	
x		ChIP-seq
	x	Flow cytometry
x		MRI-based neuroimaging

Antibodies

Antibodies used

Rat anti-LYVE-1 (cat# 14-0443-82, lot# 2494966, clone# ALY7, 1:200, IF, Invitrogen, USA), mouse anti-amyloid-β1-42 (cat# 805501, lot# B326654, clone# 12F4, 1:1000, IF, Biolegend, USA), rabbit anti-Iba1 (cat# 019-19741, lot# LEN4341, 1:500, IF, Abcam, UK), rat anti-NeuN (cat# ab279297, lot# GR3400636-6, clone# EPR12763, 1:1000, IF, Abcam, UK), rabbit anti-Syn (cat# MA5-14532, lot# WI3369463, clone# SP11, 1:100, IF, Invitrogen), mouse anti-MAP2 (cat# MA5-12826, lot# XF3603732A, clone# AP18, 1:200, IF, Invitrogen), rabbit anti-CD31 (cat# SAB4502167-100UG, lot# 210547, 1:200, IF, Sigma, USA), rat Alexa Fluor 488 rat anti-LYVE-1 (cat# 53-0443-80, lot# 2547846, clone# ALY7, 1:250, IF, Invitrogen), Alexa Fluor 488 goat anti-rat IgG (H+L) secondary antibody (cat# A-11006, lot# 2247986, 1:500, IF, Invitrogen), Alexa Fluor 488 goat anti-rabbit IgG (H+L) secondary antibody (cat# A-32731, lot# VG302077, 1:500, IF, Invitrogen), Alexa Fluor 555 goat anti-rat IgG (H+L) secondary antibody (cat# A-21434, lot# 2184321, 1:500, IF, Invitrogen), Alexa Fluor 555 goat anti-rabbit IgG (H+L) secondary antibody (cat# A-32732, lot# YA361054, 1:500, IF, Invitrogen), Alexa Fluor 633 goat anti-mouse IgG (H+L) secondary antibody (cat# A-21052, lot# 2418505, 1:500, IF, Invitrogen), rat anti-CD45-PE (cat# 12-0451-82, lot# 2356222, clone# 30-F11, 1:150, FACS, eBioscience), rat anti-CD31-FITC (cat# 11-0311-85, lot# 2373735, clone# 390, 1:100, FACS, eBioscience), hamster anti-podoplanin-PE-Cy7 (cat# 127412, lot# B355692, clone# 8.1.1, 1:100, FACS, Biolegend) and rat anti-CD11b-PerCP (cat# 101230, lot# B352939, clone# M1/70, 1:80, FACS, BioLegend).

Validation

All the antibodies used in this study were validated for the species (mouse) and application (immunofluorescence (IF) or fluorescence-activated cell sorting (FACS)) by the correspondent manufacturer and used according to supplied instructions.

IF: rat anti-LYVE-1 (cat# 14-0443-82, lot# 2494966, clone# ALY7, 1:200, IF, Invitrogen, USA), mouse anti-amyloid-β1-42 (cat# 805501, lot# B326654, clone# 12F4, 1:1000, IF, Biolegend, USA), rabbit anti-Iba1 (cat# 019-19741, lot# LEN4341, 1:500, IF, Abcam, UK), rat anti-NeuN (cat# ab279297, lot# GR3400636-6, clone# EPR12763, 1:1000, IF, Abcam, UK), rabbit anti-Syn (cat# MA5-14532, lot# WI3369463, clone# SP11, 1:100, IF, Invitrogen), mouse anti-MAP2 (cat# MA5-12826, lot# XF3603732A, clone# AP18, 1:200, IF, Invitrogen), rabbit anti-CD31 (cat# SAB4502167-100UG, lot# 210547, 1:200, IF, Sigma, USA), rat Alexa Fluor 488 rat anti-LYVE-1 (cat# 53-0443-80, lot# 2547846, clone# ALY7, 1:250, IF, Invitrogen), Alexa Fluor 488 goat anti-rat IgG (H+L) secondary antibody (cat# A-11006, lot# 2247986, 1:500, IF, Invitrogen)

FACS: rat anti-CD45-PE (cat# 12-0451-82, lot# 2356222, clone# 30-F11, 1:150, FACS, eBioscience), rat anti-CD31-FITC (cat# 11-0311-85, lot# 2373735, clone# 390, 1:100, FACS, eBioscience), hamster anti-podoplanin-PE-Cy7 (cat# 127412, lot# B355692, clone# 8.1.1, 1:100, FACS, Biolegend) and rat anti-CD11b-PerCP (cat# 101230, lot# B352939, clone# M1/70, 1:80, FACS, BioLegend).

Animals and other research organisms

Policy information about studies involving animals; ARRIVE guidelines recommended for reporting animal research, and Sex and Gender in Research

Laboratory animals

All the mice were in C57BL/6J background purchased from Jinzhihe Biotechnology Co., Ltd (Yangzhou, China). Male and female WT mice aged between 15 to 17 months served as the elderly model, and 1.5-month-old ones served as the counterpart young group. Two male AD models were used in this study: 6-month-old 5xFAD and 11-month-old APPswe/PS1ΔE9 (APP/PS1) mice, with their transgene non-carrier littermates as counterpart groups. Mice of all strains were bred under 12-h light and darkness alternating cycles with controlled temperature (20-23 °C) and humidity (50-60%), and were allowed free access to standard diet and water.

Wild animals

No wild animals were used in the study.

Reporting on sex

Male and female aged mice and male AD mice were used in the studies. Previous studies have shown that the pathology of male and female AD mice are different (Bhattacharya et al., 2014; Bouter et al., 2021) In order to avoid the influence of sex factors on the study of regulatory mechanisms, we selected male AD mice for experiments. The sex of the animals used for experiments were reported in the Results and Methods section.

Field-collected samples

No field collected samples were used in the study.

Ethics oversight

The mice treatments were performed in compliance with the Guide for the Care and Use of Laboratory Animals. All animal studies were reviewed and approved by the Institutional Animal Care and Use Committee of Hainan University (approval number: HNUAUCC-2021-00025).

Note that full information on the approval of the study protocol must also be provided in the manuscript.

Flow Cytometry

Plots

Confirm that:

☒ The axis labels state the marker and fluorochrome used (e.g. CD4-FITC).

☒ The axis scales are clearly visible. Include numbers along axes only for bottom left plot of group (a 'group' is an analysis of identical markers).

☒ All plots are contour plots with outliers or pseudocolor plots.

☒ A numerical value for number of cells or percentage (with statistics) is provided.

Methodology

Sample preparation	Mice were anesthetized and then transcardially perfused with PBS. Skullcaps were dissected and whole-mount meninges were stripped in Dulbecco's Modified Eagle Medium (DMEM, Gibco, USA), supplied with 10% FBS (Gibco), penicillin (50 U/ml, Gibco) and streptomycin (50 mg/mL, Sigma). Then meninges were incubated in DMEM containing 1 mg/ml of collagenase VIII (Sigma) and 35 U/mL of DNase I (Sigma) for 30 min at 37 °C. The digested meninges were filtrated through 70-µm nylon cell meshed for single-cell suspensions. Cells were then centrifuged (280g) at 4 °C for 10 min and resuspented in PBS. Then cells were incubated with antibodies dilutions at 4 °C for 30 min: rat anti-CD45-PE (cat# 12-0451-82, lot# 2356222, clone# 30-F11, 1:150, eBioscience), rat anti-CD31-FITC (cat# 11-0311-85, lot# 2373735, clone# 390, 1:100, eBioscience), hamster anti-podoplanin-PE-Cy7 (cat# 127412, lot# B355692, clone# 8.1.1, 1:100, Biolegend) and rat anti-CD11b-PerCP (cat# 101230, lot# B352939, clone# M1/70, 1:80, BioLegend), and finally incubated with DAPI for 5 min.
Instrument	Flow cytometry (MoFlo Astrios, Beckman Coulter, USA) was used for cell sorting.
Software	CytExpert 2.5 was used for data analysis.
Cell population abundance	CD45-cells was 79.47%.<br>Meningeal lymphatic endothelial cells was 1.10%.
Gating strategy	Meningeal lymphatic endothelial cells gated as DAPI−CD45−CD31+PDPN+.

☒ Tick this box to confirm that a figure exemplifying the gating strategy is provided in the Supplementary Information.
